# Methods for comparative effectiveness based on time to confirmed disability progression with irregular observations in multiple sclerosis

**DOI:** 10.1177/09622802231172032

**Published:** 2023-06-11

**Authors:** Thomas PA Debray, Gabrielle Simoneau, Massimiliano Copetti, Robert W Platt, Changyu Shen, Fabio Pellegrini, Carl de Moor

**Affiliations:** 1 168086 Julius Centrum voor Gezondheidswetenschappen en Eerstelijns Geneeskunde, Utrecht, Netherlands; 2 Smart Data Analysis and Statistics B.V., Utrecht, Netherlands; 3 Biogen, Toronto, Canada; 4 Fondazione IRCCS Casa Sollievo della Sofferenza, San Giovanni Rotondo, Italy; 5 Department of Epidemiology, Bioastatistics and Occupational Health, McGill University, Quebec, Canada; 6 2191 Biogen Inc, Cambridge, USA; 7 38783 Biogen Spain SL, Madrid, Spain

**Keywords:** Clustered data, comparative effectiveness, confirmed disability progression, longitudinal data, multiple sclerosis, real-world data, multiple imputation

## Abstract

Real-world data sources offer opportunities to compare the effectiveness of treatments in practical clinical settings. However, relevant outcomes are often recorded selectively and collected at irregular measurement times. It is therefore common to convert the available visits to a standardized schedule with equally spaced visits. Although more advanced imputation methods exist, they are not designed to recover longitudinal outcome trajectories and typically assume that missingness is non-informative. We, therefore, propose an extension of multilevel multiple imputation methods to facilitate the analysis of real-world outcome data that is collected at irregular observation times. We illustrate multilevel multiple imputation in a case study evaluating two disease-modifying therapies for multiple sclerosis in terms of time to confirmed disability progression. This survival outcome is derived from repeated measurements of the Expanded Disability Status Scale, which is collected when patients come to the healthcare center for a clinical visit and for which longitudinal trajectories can be estimated. Subsequently, we perform a simulation study to compare the performance of multilevel multiple imputation to commonly used single imputation methods. Results indicate that multilevel multiple imputation leads to less biased treatment effect estimates and improves the coverage of confidence intervals, even when outcomes are missing not at random.

## Introduction

Increasingly often, researchers have access to large databases with electronic healthcare records, providing information on patient characteristics, hospital admission, treatment procedures and clinical outcomes. Although these databases with real-world data (RWD) offer new opportunities to study the effectiveness of medical interventions, exposure and outcome variables are often unavailable for key study dates.^1–4^ Missing outcome data is particularly common in registries, where the measurement interval of outcome variables is irregular and tends to vary across individuals due to a lack of formalized data recording processes. The analysis of RWD therefore often requires censoring individuals with incomplete outcome data, which is problematic when outcome variables are incomplete for most individuals and may lead to bias when the censoring mechanism is informative.^5^

As an alternative to censoring, it is possible to replace missing outcomes for key study dates with a simple average or by a neighboring observation. Single imputation methods for this purpose are commonly applied in clinical trials,^6^ but may lead to biased treatment effect estimates and inflated type-I errors.^7,8^ Over the last few decades, more advanced imputation methods have been proposed that involve estimation of the (multivariate) data distribution.^9^ We here propose an extension of multilevel multiple imputation (MLMI) that can be used to recover the entire trajectory of a longitudinal variable. Motivated by challenges in comparative effectiveness research in multiple sclerosis (MS), this method is intended for situations where longitudinal data are used to define survival outcomes and are affected by irregular and informative patterns of missingness. We conduct a simulation study to compare the proposed method to existing imputation approaches. We also illustrate the imputation method in a case study comparing dimethyl fumarate (DMF) to fingolimod for treating patients with relapsing-remitting MS (RRMS).

## 2 Motivating example

MS is a chronic progressive disorder that affects approximately 2.8 million people worldwide. Several disease-modifying therapies (DMTs) are available for RRMS, the most common disease course in which patients experience episodes of disease activity, called relapses. In MS, RWD, such as MS registries, typically gather longitudinal information on patients where data are collected when the patient comes to the healthcare center for a clinical visit. Accumulating RWD on DMT usage in clinical practice opened the ground for comparative effectiveness research.

The comparative effectiveness of two DMTs is typically demonstrated using time to confirmed disability progression (CDP) as the primary or secondary outcome. Disability is characterized by the Expanded Disability Status Scale (EDSS) score, an ordinal measure taking values between 0 (no disability) and 10 (death). Disability progression is defined as an increase (e.g., 1-point) in EDSS score from an initial (e.g., baseline) EDSS measurement, provided that the increase is confirmed at a follow-up visit 3 (or 6) months later. Time to CDP is then derived as the time from baseline until a confirmed increase in EDSS score. Therefore, the definition of time to CDP depends on the ability to capture and confirm a progression given that the underlying process, EDSS score, is not measured continuously but rather only available at certain time points after baseline.

The definition of time to CDP is suitable when data are collected according to a standardized follow-up schedule but difficult to apply when the schedule for data collection is irregular. In clinical trials, follow-up visits are planned to occur every 3 months such that a disability progression observed at any given visit can always be confirmed 3 months later as per the planned schedule.^a^ In this context of standardized follow-up schedule, the ability to capture a disability progression and to confirm it is the same across patients. On the contrary, RWD data are collected when the individual comes to the healthcare center for a visit such that the timing of visits varies across and within individuals.

The ability to capture a disability progression is compromised in RWD. For example, consider two patients, one visiting his treating physician every 3 months and the other once a year. Capturing a progression for the former patient is easier as more instances of EDSS are measured compared to the latter patient. Moreover, confirming a progression is not straightforward when patients do not have a visit at the 3-month confirmation mark after their initial increase in EDSS score.

## 3 Methods

### 3.1 Notation

We consider a multicenter study where patients attend visits (e.g., clinical visit to the center) over time. Let $x_{ij}$ represent the treatment that patient *i* in center *j* received during their first visit, which takes the value 0 (control treatment) or 1 (active treatment). Further, we consider that for each patient, *M* covariates have been measured during the first visit and are recorded as $z_{mij}$ for m=1,…,M . Finally, we consider that each patient has a total of $n_{ij}^{\text{obs}}$ visits. We denote the time-varying outcome that is observed at visit *v* as $y_{vij}$ . The corresponding visit time is denoted as $t_{vij}$ . We assume that the observed patient outcomes are ordered in time, such that $t_{1ij}<t_{2ij}<\ldots <t_{n_{ij}ij}$ . This implies that $y_{1ij}$ represents the outcome that is observed at the start of treatment, with $t_{1ij}=0$ . A summary of all indices is provided in Table 1 of the Supplemental Information.

**Figure 1.**
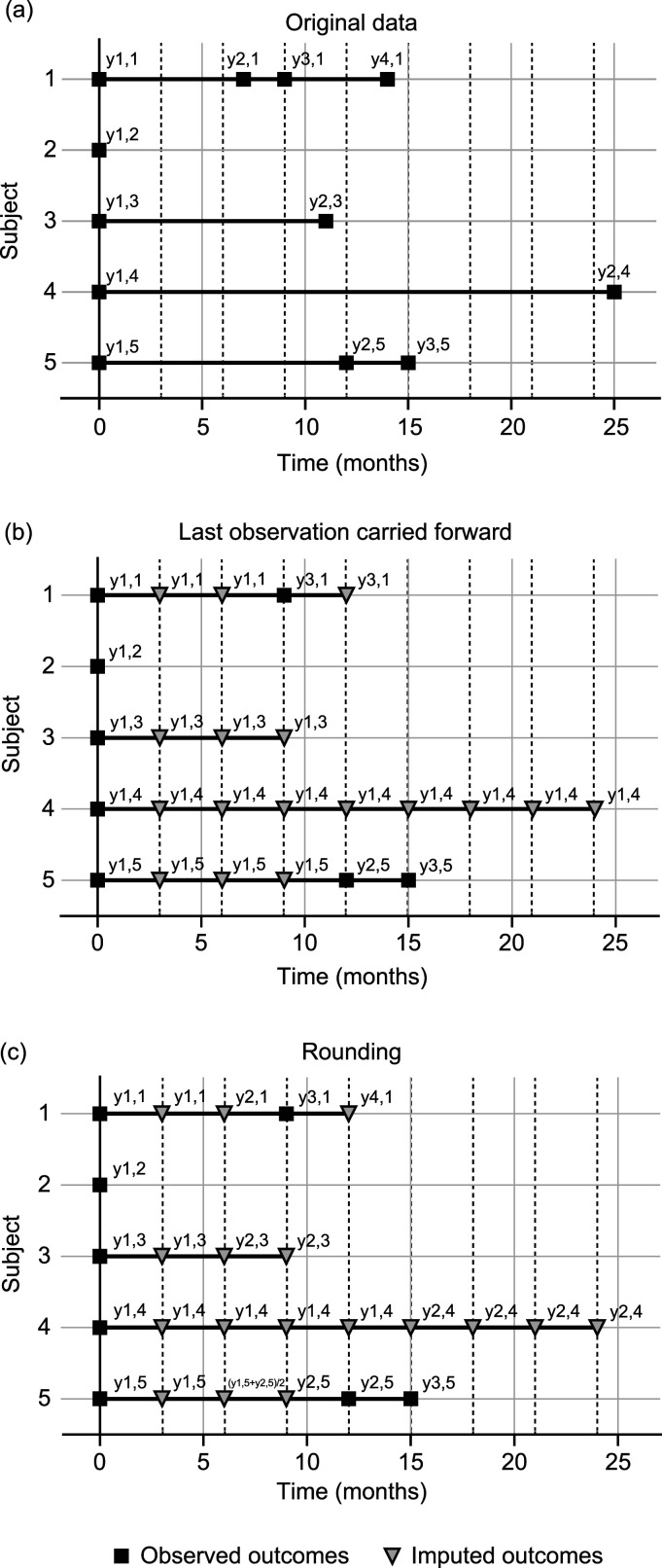
Examples of irregular visit schedules (a) and imputations on a 3-month grid using last observation carried forward (b) and rounding (c) for 5 fictive subjects. Squares and triangles represent observed and imputed outcomes, respectively.

Figure 1(a) shows irregular visits for five fictive patients superimposed on a regular schedule with visits every 3 months (the center subscript was dropped for clarity). All patients have an initial (baseline) visit $t_{1}=0$ but the visit schedule varies across patients.

### 3.2 Existing imputation methods and their limitations

Typically used methods convert the irregular visits in Figure 1(a) to a standardized schedule with equally spaced visits that allows recovering time to CDP. We describe two single imputation methods commonly used in MS: LOCF and rounding.

LOCF is one of the simplest and most common methods to account for missing data in a longitudinal analysis of repeated measures over time. It involves replacing missing patient outcomes with their most recent observation. That is, when no visit is available for time $t_{\omega }$ , the missing outcome $y_{\omega ij}^{\text{mis}}$ will be set to $y_{vij}^{\text{obs}}$ where the visit *v* is taken as $\text{max}(t_{v}^{\text{obs}}<t_{\omega }^{\text{mis}})$ , that is, the closest visit in time before visit ω . An example of LOCF is illustrated in Figure 1(b). Studies have demonstrated that LOCF analyses can lead to substantial bias, regardless of the missing data mechanism.^7^

A natural extension of LOCF is to replace missing patient outcomes at a given visit by their nearest observation in time. This method is called rounding. In contrast to LOCF, missing values for $y_{\omega ij}^{\text{mis}}$ can also be taken from patient visits where $t_{v^{\prime }}>t_{\omega }$ , that is, a visit $v^{\prime }$ that occurred after visit ω . In particular, the visit used for imputation is selected from $\max(t_{v}<t_{\omega })$ and $\text{min}(t_{v^{\prime }}>t_{\omega })$ according to $\min(t_{\omega }-\max(t_{v}<t_{\omega }),\;\min(t_{v^{\prime }}>t_{\omega })-t_{\omega })$ , that is, the visit *v* or $v^{\prime }$ which is closest in time to visit ω . An illustration of rounding is provided in Figure 1(c).

Finally, a third strategy is to combine LOCF and rounding, and to replace missing patient outcomes with the average of the observed outcomes closest in time before the missing visit and after the missing visit (henceforth AVG).

**Figure 2.**
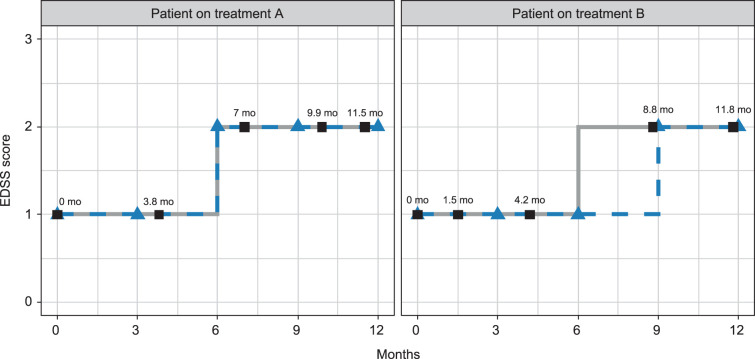
Expanded Disability Status Scale (EDSS) underlying trajectories (solid lines) and observed trajectories (dashed lines) for two patients on treatments A and B. The black squares are EDSS scores observed on an irregular visit schedule which differs between the two patients. The blue triangles are the observed EDSS mapped to a regular 3-month grid. mo: months.

We use a toy example to illustrate how rounding can lead to misleading results when comparing two treatments on their ability to slow disability progression in the MS context. Consider data measured on two individuals receiving either treatment A or B (see Figure 2). For simplicity, assume that both individuals have similar prognosis and were equally likely to receive one treatment or the other such that any difference in disability progression is attributable to the treatment only. Further assume that both treatments are equivalent in terms of their ability to slow disease progression such that the two individuals have the same underlying EDSS trajectory (solid gray lines), with CDP occurring 6 months after treatment initiation. However, the two individuals follow irregular visit schedules such that their EDSS trajectories are observed at different times (black squares). With rounding, the observed EDSS scores are mapped to a standardized 3-month grid (blue triangles). This leads to both individuals having similar rounded EDSS trajectories (dashed blue lines) except at 6 months when the individual on treatment A is attributed an EDSS value of 2 (using the observed EDSS at 7 months) while the one on treatment B is attributed a value of 1 (based on the observed EDSS at 4.2 months). Consequently, the time to CDP is 6 months on treatment A compared to 9 months on treatment B, mistakenly suggesting that treatment B slows disability progression. In this toy example, the difference in observed time to CDP is entirely due to differences in visit schedules between individuals on treatment A and B.

As an alternative to aforementioned single imputation methods, it is possible to adopt multiple imputation methods that better account for uncertainty in imputed values. For example, when dealing with time-to-event outcomes such as CDP, substantive-model-compatible multiple imputations could be used to recover missing values.^10^ Unfortunately, because disease progression can rarely be confirmed in RWD, the lack of outcome events severely hampers the implementation of the substantive (survival) model. It, therefore, seems more promising to adopt imputation methods that recover the longitudinal EDSS outcomes conditional on individual characteristics. Huque et al.^9^ provide a comprehensive overview of multiple imputation methods for this purpose. These methods consider repeated measurements of time-dependent variables as distinct variables or impute each incomplete time-dependent variable separately using hierarchical models.^11–13^ The former approach can only be used when data are collected at fixed time intervals and is therefore not suitable to recover CDP outcomes. Although hierarchical models seem more promising for imputing irregularly spaced data, existing methods have several limitations.

First of all, CDP outcomes are defined using repeated measurements of the EDSS score and require considering their entire sequence. Existing imputation methods typically assume that repeated observations are independent once clustering has been taken into account. In practice, more complex correlation structures are needed to allow for dependencies between repeated observations. A second limitation of existing multilevel imputation methods is that they usually allow for only one level of clustering. It is not uncommon that longitudinal RWD is collected from multiple practices, hospitals, or health care centers, and therefore requires the implementation of three-level imputation models.^14^ However, such methods are not always available in mainstream statistical software. Finally, and perhaps most importantly, imputation methods typically assume that data are missing at random. This assumption is extremely unrealistic for EDSS outcomes because patient visits to the clinic are often determined by changes in patient characteristics, physician's preference, or treatment status.^2,15^ For this reason, we consider an extension to multilevel imputation methods that can accommodate for aforementioned limitations.

### 3.3 Modeling the full underlying continuous-time process

Similar to existing multilevel imputation methods, we propose to estimate the outcome trajectories of all individuals by adopting multilevel (hierarchical) models.^16,17^ These longitudinal methods have several advantages. First, they account for the multiple repeated measures nested within each individual and within each cluster (e.g., healthcare institution). Second, they do not require observations at rigidly fixed intervals (as is rarely the case in clinical practice). Third, they allow accounting for important covariates, such as confounders, prognostic factors, or predictors of treatment effect. Our imputation model adopts the following equation to describe the longitudinal outcome (*Y*) as a function of the received treatment (*X*), the treatment exposure time (*T*), and the baseline covariates (***Z***):

(1) $$\begin{array}{r} y_{vij}=\alpha +a_{i}+b_{j}+\overset{K}{\underset{k=1}{\sum }}\;\beta_{k}f_{k}(t_{vij})+\overset{L}{\underset{l=1}{\sum }}\;\delta_{l}g_{l}(t_{vij},\;x_{ij})+\overset{M}{\underset{m=1}{\sum }}\;\gamma_{m}z_{mij}+\varepsilon_{vij} \end{array}$$

with $a_{i}∼N(0,\;\tau_{0}^{2})$ , $b_{j}∼N(0,\;\;\tau_{1}^{2})$ , and $\varepsilon_{ij}∼MVN(0,\;\Sigma_{ij})$ .

In the context of MS and CDP, $y_{vij}$ denotes the observed EDSS score for individual *i* in center *j* at visit *v* (with corresponding visit time $t_{vij}$ ). The intercept α represents the average EDSS when initiating treatment when all the baseline covariates (***Z***) equal zero. Individual- and center-specific intercepts are allowed through $a_{i}$ and $b_{j}$ , respectively. The function $f_{k}$ and associated coefficients $\beta_{k}$ define the effect of time on EDSS trajectories. For instance, we may adopt a linear relationship between time and EDSS scores by specifying $\sum_{k=1}^{K}\beta_{k}t_{vij}^{k}$ and setting K=1 . For non-linear relationships, we can set K≥2 . The function $g_{l}$ and associated coefficients $\delta_{l}$ define the effect of treatment and its interaction with time. It may also be expressed as a polynomial $\sum_{l=1}^{L}\delta_{l}x_{ij}t_{vij}^{l}$ , and setting L=1 would lead to $\delta_{l}$ representing the overall treatment effect. Higher-order polynomials for $g_{l}$ may be appropriate if the treatment effect changes (e.g., attenuates) over time. We here define $t_{1ij}=0$ as the baseline visit and assume that the treatment effect at $t_{1ij}$ is 0. Finally, because EDSS scores are often related to (changes in) individual characteristics, model (1) considers the adjustment of *M* prognostic factors and/or confounders. For simplicity, we assume that the prognostic factors $z_{mij}$ are time-invariant and that corresponding effects do not vary across centers. These assumptions may be relaxed, as illustrated by Erler.^18^

The residuals $\varepsilon_{vij}$ capture the error terms across visits, individuals, and centers. The vector $\epsilon_{ij}=(\epsilon_{1ij},\;\ldots ,\;\;\epsilon_{n_{ij}ij}{)}^{T}$ regroups the errors over time for individual *i*. We assume that the errors of a given individual are normally distributed around 0 with variance–covariance matrix $\Sigma_{ij}$ . Autocorrelation is present when the off-diagonal entries of $\Sigma_{ij}$ differ from zero. Further, we allow for dependence between EDSS scores taken on the same individual over time by implementing an autocorrelation structure.^19,20^ Importantly, the autocorrelation must account for the fact that the EDSS scores are measured at unequally spaced visits such that the time elapsed between visits $t_{vij}$ and $t_{(v+1)ij}$ can differ both across and within individual over time. As such, a simple autoregressive-1 (AR1) structure that ignores the actual timing of the visits would be inappropriate. One natural approach is to assume that the correlation between two within-individual errors $\varepsilon_{\zeta ij}$ and $\varepsilon_{\xi ij}$ depends on some distance between their observation times $t_{\zeta ij}$ and $t_{\xi ij}$ . For example, we may consider that the correlation between two EDSS scores measured on the same individual decreases as the time elapsed between the corresponding visit times increases. To this purpose, we adopt an exponential spatial correlation structure,^20^ where the correlation between two within-individual errors is given as $\text{cor}(\varepsilon_{\zeta ij},\;\varepsilon_{\xi ij})=\text{exp}(-|t_{\zeta ij}-t_{\xi ij}|/d)$ . We then have:

(2) $$\begin{array}{r} \Sigma_{ij}=\left[\begin{array}{cccc} \sigma^{2} & \text{exp}\left(-\frac{\left|t_{1ij}-t_{2ij}\right|}{d}\right)\sigma^{2} & \ldots & \text{exp}\left(-\frac{\left|t_{1ij}-t_{n_{ij}ij}\right|}{d}\right)\sigma^{2}\\ \text{exp}\left(-\frac{\left|t_{1ij}-t_{2ij}\right|}{d}\right)\sigma^{2} & \sigma^{2} & \ldots & \text{exp}\left(-\frac{\left|t_{2ij}-t_{n_{ij}ij}\right|}{d}\right)\sigma^{2}\\ \vdots & & \ddots & \vdots \\ \text{exp}\left(-\frac{\left|t_{1ij}-t_{n_{ij}ij}\right|}{d}\right)\sigma^{2} & \ldots & \ldots & \sigma^{2} \end{array}\right] \end{array}$$

Note that each individual has a distinct $n_{ij}$ -dimensional error covariance matrix $\Sigma_{ij}$ with shared parameters σ and *d*.

When visit times do not depend on unobserved covariates that also affect $y_{vij}$ , model (1) can reliably be estimated with the observed data. Estimates for the regression coefficients can then directly be used for statistical inference. Strictly speaking, imputation may therefore not always be necessary. Indeed, many longitudinal studies consider missing outcome data as ignorable and do not make any attempts for their recovery. Problems, however, arise when model (1) does not address the research question, or when $y_{vij}$ represents a longitudinal covariate (rather than an outcome variable).

We, therefore, develop a new class of one-stage imputation methods that can be used to impute longitudinal trajectories in large data sets with clustering. We build upon the theory of multivariate imputation by chained equations (MICE), and adopt an (approximate) Bayesian approach for drawing parameters in the imputation model.^21,22^

#### 3.3.1 Approximate Bayesian approach

We define the vector of observable outcomes for individual *i* in center *j* as $y_{ij}=\{y_{ij}^{\text{obs}},\;y_{ij}^{\text{mis}}\}$ with corresponding visit times $t_{ij}=\{t_{ij}^{\text{obs}},\;t_{ij}^{\text{mis}}\}$ . The total number of observable time points is then given as $n_{ij}=n_{ij}^{\text{obs}}+n_{ij}^{\text{mis}}$ . For example, consider an individual with a visit at treatment start (EDSS = 3), and another visit 2.5 years later (EDSS = 4). We thus have a total of $n_{ij}^{\text{obs}}=2$ visits with visit times $t_{ij}^{\text{obs}}=\{0,\;\;30\}$ months and corresponding EDSS scores $y_{ij}^{\text{obs}}=\{3,\;\;4\}$ . If we are interested in recovering EDSS scores on a 6-month grid for up to 2 years, then $n_{ij}^{\text{mis}}=4$ with $t_{ij}^{\text{mis}}=(6,\;\;12,\;\;18,\;\;24)$ months and corresponding missing EDSS scores $y_{ij}^{\text{mis}}=\{?,\;\;?,\;\;?,\;\;?\}$ . Below, we describe an algorithm to generate random draws for $y_{ij}^{\text{mis}}$ from their posterior distribution.

First, we estimate the model parameters $\hat{\theta }=\{\hat{\alpha },\;{\hat{\beta }}_{1},\;\ldots ,\;{\hat{\beta }}_{K},\;\;{\hat{\delta }}_{1},\;\ldots ,\;{\hat{\delta }}_{L},\;{\hat{\gamma }}_{1},\;\ldots ,\;{\hat{\gamma }}_{M},\;\ldots ,\;\;{\hat{\tau }}_{0},\;{\hat{\tau }}_{1},\;\hat{\sigma },\;\;\hat{d}\}$ and their variance covariance matrix $S_{\theta }$ by maximizing the restricted log-likelihood of model (1) fitted to the available data with a total of $n_{ij}^{\text{obs}}$ observations. Although it is possible to allow for correlation between all entries of $\hat{\theta }$ , many software packages do not report the covariance between ${\hat{\theta }}_{\text{f}}=\{\hat{\alpha },\;\hat{\beta },\;\;\hat{\gamma },\;\hat{\delta },\;\hat{\sigma }\}$ , ${\hat{\theta }}_{\text{h}}=\{{\hat{\tau }}_{0},\;{\hat{\tau }}_{1}\}$ and ${\hat{\theta }}_{\text{d}}=\{\hat{d}\}$ . For this reason, we here consider that $S_{\theta }$ is a block diagonal matrix. We can then directly draw from the posterior distribution of $\theta_{\text{h}}$ . We adopt the parameterization of Pinheiro and Bates^20^ as implemented in the R package *nlme*, and generate random samples for $\tau_{0}$ and $\tau_{1}$ according to:

$$\begin{array}{r} \begin{array}{c} \log\;\tau_{0}^{*}∼N(\log\;{\hat{\tau }}_{0},\;\;\text{var}(\log\;{\hat{\tau }}_{0}))\\ \log\;\tau_{1}^{*}∼N(\log\;{\hat{\tau }}_{1},\;\;\text{var}(\log\;{\hat{\tau }}_{1})) \end{array} \end{array}$$

The total random effects variance of the intercept terms is then given by $\tau^{2*}=\tau_{0}^{2*}+\tau_{1}^{2*}$ . In a similar fashion, we generate an independent random draw for the range of the exponential correlation structure:

$$\begin{array}{r} \log\;d^{*}∼N(\log\;\hat{d},\;\;\text{var}(\log\;\hat{d})) \end{array}$$

Further, if we apply the standard prior distributions $\text{Pr}(\theta_{\text{f}})\propto \sigma^{-2}$ , we can also generate a random draw for $\sigma^{2}$ according to:

$$\begin{array}{r} \sigma^{2*}∼\;\omega ({\hat{\sigma }}^{2}\chi_{df}^{-2}) \end{array}$$

where $\chi_{\omega }^{2}$ is a chi-squared distribution with $\omega =n_{ij}^{\text{obs}}-1-K-L-M$ degrees of freedom. For the fixed effects, we have:

$$\begin{array}{r} (\alpha ,\;\beta ,\;\gamma ,\;\delta {)}^{*}∼\text{MVN}\left((\hat{\alpha },\;\hat{\beta },\;\hat{\gamma },\;\hat{\delta }),\;\;\text{var}(\hat{\alpha },\;\hat{\beta },\;\hat{\gamma },\;\hat{\delta })\frac{\sigma^{2*}}{{\hat{\sigma }}^{2}}\right) \end{array}$$

#### 3.3.2 Hierarchical imputation of missing responses

After drawing $\tau^{2*}$ , $\sigma^{2*}$ , $d^{*}$ and $(\alpha ,\;\beta ,\;\gamma ,\;\delta {)}^{*}$ , we can generate imputations for EDSS scores at arbitrary visit times. First, we need to obtain the random effects $a_{i}^{*}$ and $b_{j}^{*}$ for individuals for whom imputations are required, that is, individuals for whom $t_{ij}^{\text{mis}}\neq \emptyset$ . However, rather than drawing $a_{i}^{*}$ and $b_{j}^{*}$ separately, we directly draw $q_{ij}^{*}=a_{i}^{*}+b_{j}^{*}$ from its posterior distribution $N({\tilde{q}}_{ij},\;{\tilde{\varsigma }}_{ij}^{2})$ , where

$$\begin{array}{r} {\tilde{q}}_{ij}=(\tau^{2*}1_{n_{ij}^{\text{obs}}}^{\text{T}}){\left(\tau^{2*}1_{n_{ij}^{\text{obs}}}1_{n_{ij}^{\text{obs}}}^{\text{T}}+\Sigma_{ij}^{*,\text{obs}}\right)}^{-1}(y_{ij}^{obs}-{\hat{y}}_{ij}^{*,\text{obs}})\\ {\tilde{\varsigma }}_{ij}^{2}=\tau^{2*}-(\tau^{2*}1_{n_{ij}^{\text{obs}}}^{\text{T}}){\left(\tau^{2*}1_{n_{ij}^{\text{obs}}}1_{n_{ij}^{\text{obs}}}^{\text{T}}+\Sigma_{ij}^{*,\text{obs}}\right)}^{-1}(\tau^{2*}1_{n_{ij}^{\text{obs}}}) \end{array}$$

In this expression, $1_{n_{ij}^{\text{obs}}}$ is a $n_{ij}^{\text{obs}}\times 1$ vector of ones. The $n_{ij}^{\text{obs}}\times \text{\textbackslash{};}n_{ij}^{\text{obs}}$ within-individual covariance matrix is derived as follows:

$$\begin{array}{r} \Sigma_{ij}^{*,\text{obs}}=\left[\begin{array}{cccc} \sigma^{*2} & \text{exp}\left(-\frac{\left|t_{1ij}^{\text{obs}}-t_{2ij}^{\text{obs}}\right|}{d^{*}}\right)\sigma^{*2} & \;\ldots \;\; & \text{exp}\left(-\frac{\left|t_{1ij}^{\text{obs}}-t_{n_{ij}^{\text{obs}}ij}\right|}{d^{*}}\right)\sigma^{*2}\\ \text{exp}\left(-\frac{\left|t_{1ij}^{\text{obs}}-t_{2ij}^{\text{obs}}\right|}{d^{*}}\right)\sigma^{*2} & \sigma^{*2} & \;\ldots \;\; & \text{exp}\left(-\frac{\left|t_{2ij}^{\text{obs}}-t_{n_{ij}^{\text{obs}}ij}^{\text{obs}}\right|}{d^{*}}\right)\sigma^{*2}\\ \vdots & & \ddots & \vdots \\ \text{exp}\left(-\frac{\left|t_{1ij}^{\text{obs}}-t_{n_{ij}^{\text{obs}}ij}^{\text{obs}}\right|}{d^{*}}\right)\sigma^{*2} & \ldots & \ldots & \sigma^{*2} \end{array}\right] \end{array}$$

Further, the $n_{ij}^{\text{obs}}$ fixed effects predictions for $y_{ij}^{\text{obs}}$ at visit times $t_{ij}^{\text{obs}}$ are denoted as ${\hat{y}}_{ij}^{*,\text{obs}}$ with entries:

$$\begin{array}{r} \text{\textbackslash{};}{\hat{y}}_{ij}^{*,\text{obs}}=\alpha^{*}+\overset{K}{\underset{k=1}{\sum }}\;\beta_{k}^{*}f_{k}(t_{vij}^{\text{obs}})+\overset{L}{\underset{l=1}{\sum }}\;\delta_{l}^{*}g_{l}(t_{vij}^{\text{obs}},\;x_{ij})+\overset{M}{\underset{m=1}{\sum }}\;\gamma_{m}^{*}z_{mij} \end{array}$$

An imputation for the vector $y_{ij}^{\text{mis}}$ can now be generated as follows:

(3) $$\begin{array}{rl} y_{ij}^{*,\text{mis}} & =\alpha^{*}1_{n_{ij}^{\text{mis}}}+q_{ij}^{*}1_{n_{ij}^{\text{mis}}}+\overset{K}{\underset{k=1}{\sum }}\;f_{k}(t_{ij}^{\text{mis}})\beta_{k}^{*}\\ & \;+\overset{L}{\underset{l=1}{\sum }}\;f_{l}(t_{ij}^{\text{mis}},\;\;x_{ij}1_{n_{ij}^{\text{mis}}})\delta_{l}^{*}+\overset{M}{\underset{m=1}{\sum }}\;z_{mij}1_{n_{ij}^{\text{mis}}}\gamma_{m}^{*}+\epsilon_{ij}^{*,\text{mis}} \end{array}$$

It is common to draw the vector with residual errors $\epsilon_{ij}^{*,\text{mis}}$ from a multivariate normal distribution with zero mean and covariance $\sigma^{2*}\text{\textbackslash{};I}(n_{ij}^{\text{mis}})$ . However, because the longitudinal outcomes of an individual are rarely independent, we propose to account for potential autocorrelation. Hereto, we first calculate the residual errors $\epsilon_{vij}^{*\text{, obs}}=y_{vij}^{\text{obs}}-{\hat{y}}_{vij}^{*,\text{obs}}-q_{ij}^{*}$ for all observed visit times. Since it is assumed that $\left(\epsilon_{ij}^{*,\text{mis}},\;\epsilon_{ij}^{*,\text{obs}})\;∼\;MVN(0,\;\;\Sigma_{ij}^{*}\right)$ , the conditional distribution $\text{Pr}(\epsilon_{ij}^{*,\text{mis}}|\epsilon_{ij}^{*,\text{obs}})$ is also normal, with:

$$\begin{array}{r} \text{E}(\epsilon_{ij}^{*,\text{mis}}|\epsilon_{ij}^{*,\text{obs}})=C_{ij}^{*}{\left(\Sigma_{ij}^{*,\text{obs}}\right)}^{-1}\epsilon_{ij}^{*,\text{obs}}\\ \text{var}(\epsilon_{ij}^{*,\text{mis}}|\epsilon_{ij}^{*,\text{obs}})=\Sigma_{ij}^{*,\text{mis}}-C_{ij}^{*}{\left(\Sigma_{ij}^{*,\text{obs}}\right)}^{-1}C{{}_{ij}^{*}}^{\text{T}} \end{array}$$

In these expressions, $C_{ij}^{*}$ is a $n_{ij}^{\text{mis}}\times n_{ij}^{\text{obs}}$ matrix with the following covariance entries:

$$\begin{array}{r} {\text{C}}_{ij}^{*}=\left[\begin{array}{ccc} \text{exp}\left(-\frac{\left|t_{1ij}^{\text{mis}}-t_{1ij}^{\text{obs}}\right|}{d^{*}}\right)\sigma^{*2} & \ldots & \text{exp}\left(-\frac{\left|t_{1ij}^{\text{mis}}-t_{n_{ij}^{\text{obs}}ij}^{\text{obs}}\right|}{d^{*}}\right)\sigma^{*2}\\ \text{exp}\left(-\frac{\left|t_{2ij}^{\text{mis}}-t_{1ij}^{\text{obs}}\right|}{d^{*}}\right)\sigma^{*2} & \ldots & \text{exp}\left(-\frac{\left|t_{2ij}^{\text{mis}}-t_{n_{ij}^{\text{obs}}ij}^{\text{obs}}\right|}{d^{*}}\right)\sigma^{*2}\\ \vdots & \ddots & \vdots \\ \text{exp}\left(-\frac{\left|t_{n_{ij}^{\text{mis}}ij}^{\text{mis}}-t_{1ij}^{\text{obs}}\right|}{d^{*}}\right)\sigma^{*2} & \ldots & \text{exp}\left(-\frac{\left|t_{n_{ij}^{\text{mis}}ij}^{\text{mis}}-t_{n_{ij}^{\text{obs}}ij}^{\text{obs}}\right|}{d^{*}}\right)\sigma^{*2} \end{array}\right] \end{array}$$

and where $\Sigma_{ij}^{*,\text{mis}}$ is:

$$\begin{array}{r} \Sigma_{ij}^{*,\text{mis}}=\left[\begin{array}{cccc} \sigma^{*2} & \text{exp}\left(-\frac{\left|t_{1ij}^{\text{mis}}-t_{2ij}^{\text{mis}}\right|}{d^{*}}\right)\sigma^{*2} & \ldots & \text{exp}\left(-\frac{\left|t_{1ij}^{\text{mis}}-t_{n_{ij}^{\text{mis}}ij}^{\text{mis}}\right|}{d^{*}}\right)\sigma^{*2}\\ \text{exp}\left(-\frac{\left|t_{1ij}^{\text{mis}}-t_{2ij}^{\text{mis}}\right|}{d^{*}}\right)\sigma^{*2} & \sigma^{*2} & \ldots & \text{exp}\left(-\frac{\left|t_{2ij}^{\text{mis}}-t_{n_{ij}^{\text{mis}}ij}^{\text{mis}}\right|}{d^{*}}\right)\sigma^{*2}\\ \vdots & & \ddots & \vdots \\ \text{exp}\left(-\frac{\left|t_{1ij}^{\text{mis}}-t_{n_{ij}^{\text{mis}}ij}^{\text{mis}}\right|}{d^{*}}\right)\sigma^{*2} & \ldots & \ldots & \sigma^{*2} \end{array}\right] \end{array}$$

When implementing equation (3) to impute the missing EDSS scores, it is recommended to generate multiple imputed data sets. The imputed data sets are then analysed separately (e.g., to derive time to CDP and estimate the treatment effect), after which the corresponding results (e.g., log hazard ratio) are pooled using Rubin's rules.^23^

Note that equation (3) yields imputed values that are continuous, and therefore do not necessarily reflect realistic EDSS scores. One possible solution is to round individual draws for $y_{ij}^{*,\text{mis}}$ to the nearest half-integer and to truncate corresponding values between 0 and 9.5. However, a more advanced approach is to consider predictive mean matching (PMM).^24^

#### 3.3.3 Predictive mean matching

We adopt the following PMM algorithm to generate more realistic imputations for the unobserved EDSS scores $y_{ij}^{\text{mis}}$ :

1. Draw $\tau^{2*}$ , $\sigma^{2*}$ and $(\alpha ,\;\beta ,\;\gamma ,\;\delta {)}^{*}$ and $q_{ij}^{*}$ using the aforementioned procedure.
2. Calculate ${\hat{y}}_{vij}^{\text{obs}}=\hat{\alpha }+{\hat{a}}_{i}+{\hat{b}}_{j}+\overset{K}{\underset{k=1}{\sum }}\;{\hat{\beta }}_{k}f_{k}(t_{vij}^{\text{obs}})+\overset{L}{\underset{l=1}{\sum }}\;{\hat{\delta }}_{l}f_{l}(t_{vij}^{\text{obs}},\;x_{ij})+\overset{M}{\underset{m=1}{\sum }}\;{\hat{\gamma }}_{m}z_{mij}$ for all patients and all $n_{ij}^{\text{obs}}$ observed visit times. The corresponding vector is denoted as ${\hat{y}}^{\text{obs}}$ .
3. Calculate the vector ${\hat{y}}_{ij}^{\text{mis}}=\alpha^{*}+q_{ij}^{*}+\overset{K}{\underset{k=1}{\sum }}\;\beta_{k}^{*}f_{k}(t_{ij}^{\text{mis}})+\overset{L}{\underset{l=1}{\sum }}\;\delta_{l}^{*}f_{l}(t_{ij}^{\text{mis}},\;x_{ij})+\overset{M}{\underset{m=1}{\sum }}\;\gamma_{m}^{*}z_{mij}+C_{ij}^{*}(\Sigma_{ij}^{*\text{, obs}}{)}^{-1}\epsilon_{ij}^{*\text{, obs}}$ , which provides the most likely values for $y_{ij}^{\text{mis}}$ given the random parameter drawn from step 1 and the observed residual errors in $y_{ij}^{\text{obs}}$ .
4. For each $\varphi \in t_{ij}^{\text{mis}}$ calculate $\Delta =|{\hat{y}}^{\text{obs}}-{\hat{y}}_{\varphi ij}^{\text{mis}}|$ .
5. Randomly sample one value from ( $\Delta_{\left(1\right)}$ , … , $\Delta_{\left(P\right)}$ ) where $\Delta_{\left(1\right)}$ , … , $\Delta_{\left(P\right)}$ are the *P* smallest elements in Δ , respectively, and take the corresponding $y_{vij}^{\text{obs}}$ as the imputation.

In case multiple imputations are required, repeat steps 1–5, each time saving the completed data set. The resulting imputation algorithm has been implemented in R and is freely available from Github.

## 4 Simulation study

We conduct an extensive simulation study to compare the three approaches for dealing with informative missing outcome data. We consider a multicenter study where individuals are treated with one of two DMTs. The outcome is time to CDP defined as a function of the EDSS score recorded at each visit. To mimic the irregular visit schedules in clinical practice, visits were deleted according to an informative missingness procedure. That is, for some scenarios, we consider that EDSS scores are missing not at random.

### 4.1 Data-generating mechanism

We consider 20 clinical centers with 500 individuals each. Individuals receive DMT A or DMT B according to a nonrandomized treatment allocation and are followed for a maximum of 60 months. Data were generated according to an existing observational cohort of individuals with RRMS in clinical practice settings in Europe, Australia, Argentina, and Canada,^25^ such that the baseline EDSS scores have a mean value of 3.45 and a standard deviation of 1.64 (Supplemental Information A2.1). The EDSS score for each individual is observable on a monthly basis and defined by an individual-specific intercept (a random effect to personalize disease severity at treatment start), a center-specific intercept (a random effect to introduce between-center heterogeneity in baseline disease severity), a time effect (to mimic an annual EDSS increase of 0.168),^26^ a treatment effect, and an effect of age at treatment start. We consider three types of treatment effect: (a) Treatment does not affect disease progression, (2) DMT B decreases annual EDSS progression from 0.168 to 0.084 (moderate treatment effect), and (3) DMT B stops annual EDSS progression (strong treatment effect). For each individual, we generated 61 EDSS scores that reflect hypothetical observations in monthly intervals. Subsequently, each score is rounded to the nearest half-integer and truncated between 0 and 9.5. Note that this transformation implies that EDSS scores are no longer normally distributed, which may affect the validity of MLMI. Details of the data-generating mechanism are reported in the Supplemental Information.

We subsequently generated irregular visit patterns by randomly setting some of the 61 generated EDSS scores as missing, except for the baseline EDSS score, which was always observed. To this purpose, we consider six patterns of missingness where the visit probability for each patient varies according to their MS center, received treatment and/or unobserved EDSS scores (Table 1). The latter scenario mimics situations where EDSS scores are missing not at random. Technical details of each visit pattern and an illustration of them are provided in the Supplemental Information.

### 4.2 Data analysis and evaluation

In total, we consider 18 scenarios (6 visit patterns * 3 types of treatment effect). For each scenario, a total of 500 datasets were generated with 61 (observable) EDSS scores per individual. After introducing missing values in the monthly EDSS scores, five strategies were applied to recover the EDSS scores on a 3-month grid between baseline and the last visit: LOCF, rounding, AVG, multilevel imputation with EDSS conversion multilevel imputation with rounding (MLMI-RND), and multilevel imputation with PMM (MLMI-PMM). For the last two approaches, we generated 20 imputed datasets.

**Table 1.** Simulated scenarios.

			Variables affecting the visit pattern				Average visit count	
Scenario	Treatment effect^a^	Visit pattern	Center	Baseline EDSS	Treatment	Current EDSS	DMT A	DMT B
1	None	1	Yes	—	—	—	8.5	8.5
2	Moderate	1	Yes	—	—	—	8.5	8.5
3	Strong	1	Yes	—	—	—	8.5	8.5
4	None	2	—	Yes	—	—	7.8	8.5
5	Moderate	2	—	Yes	—	—	7.8	8.5
6	Strong	2	—	Yes	—	—	7.8	8.5
7	None	3	—	—	Yes^b^	—	11	6.6
8	Moderate	3	—	—	Yes**^b^**	—	11	6.6
9	Strong	3	—	—	Yes^b^	—	11	6.6
10	None	4	—	—	Yes^c^	—	9.2	5.9
11	Moderate	4	—	—	Yes^c^	—	9.2	5.9
12	Strong	4	—	—	Yes^c^	—	9.2	5.9
13	None	5	Yes	—	Yes	—	11.3	6.6
14	Moderate	5	Yes	—	Yes	—	11.3	6.6
15	Strong	5	Yes	—	Yes	—	11.3	6.6
16	None	6	—	—	Yes	Yes	6.9	10.6
17	Moderate	6	—	—	Yes	Yes	6.9	11.3
18	Strong	6	—	—	Yes	Yes	6.9	12.2

EDSS: Expanded Disability Status Scale; DMT: disease-modifying therapy.

^a^ None: beta = 0, moderate: beta = −0.007, strong: beta = −0.014.

^b^ Individuals receiving DMT A have a visit every 6 months, whereas individuals receiving DMT B have a visit every 9 months.

^c^ Individuals receiving DMT A have a visit every 3 months, whereas individuals receiving DMT B have a visit every 9 months.

After imputation, the longitudinal EDSS scores were converted into time to CDP, where CDP is defined as a ≥0.5 point increase in EDSS from baseline EDSS ≥6.0 , a ≥1.0 point increase from baseline EDSS between 1.0 and 5.5, or a ≥1.5 point increase in EDSS from a baseline EDSS below 1. The increase must be observed at a follow-up visit 3 months later to confirm the progression. We then study the marginal treatment effect on time to CDP using a Cox regression model with stabilized inverse probability of treatment weights and a baseline hazard stratified by center. We adopt a corrected sandwich estimator to obtain robust estimates of the standard errors (SEs).^27^ We specify time to CDP as a function of treatment, age, and baseline EDSS score. As a final step, the estimated log hazard ratios were pooled across the imputed datasets using Rubin’s rules.

We compare the three imputation methods in terms of three performance criteria across the 500 simulation runs: (a) the root mean squared error (RMSE) of the imputed EDSS scores across all visits, (b) the estimated hazard ratios for treatment in terms of average bias and RMSE, and (c) the coverage of estimated hazard ratios. An overview of all scenarios is summarized in Table 1.

### 4.3 Results

Simulation study results for all 18*500 generated datasets with 10,000 individuals each are depicted in the Supplemental Information (A3). Figure S3 indicates that the RMSE of imputed EDSS scores is highest when adopting LOCF, and lowest when adopting MLMI-PMM, regardless of the true underlying treatment effect. For example, in the scenarios where the visit pattern depends on center only (visit pattern 1), the RMSE decreases from 0.61 (LOCF) to 0.48 (MLMI-PMM), which corresponds to a relative improvement of 21%. Conversely, when other imputation methods were applied, the RMSE ranged from 0.49 (MLMI-RND) to 0.53 (rounding). These differences remained consistent across all 18 evaluated scenarios, even when EDSS scores were missing not at random (MNAR) (scenarios 16–18).

The RMSE of estimated hazard ratios was highest when adopting LOCF, and lowest when adopting multilevel imputation with PMM (Table 2). Results in the Supplemental Information (Figure S4) indicate that these discrepancies mainly arise due to bias. For example, in scenario 16 where EDSS scores are MNAR and both treatments are equally effective, the average treatment effect estimate varies between 1.12 (LOCF), 1.07 (rounding), and 1.00 (MLMI-PMM). This corresponds to a bias of 0.12 for LOCF (where RMSE = 0.13), a bias of 0.07 for rounding (where RMSE = 0.08) and zero bias for multilevel imputation (where RMSE = 0.03).

**Table 2.** Simulation results.

			RMSE of estimated HR				Coverage of 95% CI			
Scenario	Visit pattern	Treatment effect	LOCF	RND	MLMI-RND	MLMI-PMM	LOCF	RND	MLMI-RND	MLMI-PMM
1	1	None	0.029	0.027	0.023	0.024	92%	95%	98%	96%
2	1	Moderate	0.057	0.043	0.031	0.019	31%	50%	76%	96%
3	1	Strong	0.075	0.056	0.038	0.018	1%	8%	32%	90%
4	2	None	0.033	0.029	0.023	0.025	92%	92%	99%	97%
5	2	Moderate	0.073	0.051	0.051	0.028	22%	47%	35%	86%
6	2	Strong	0.093	0.062	0.064	0.031	0%	6%	1%	60%
7	3	None	0.198	0.135	0.071	0.024	0%	1%	26%	96%
8	3	Moderate	0.088	0.122	0.065	0.018	0%	0%	10%	95%
9	3	Strong	0.029	0.101	0.052	0.013	47%	0%	8%	98%
10	4	None	0.093	0.043	0.056	0.030	7%	73%	51%	92%
11	4	Moderate	0.022	0.023	0.065	0.021	88%	91%	11%	93%
12	4	Strong	0.032	0.035	0.061	0.018	54%	46%	1%	91%
13	5	None	0.152	0.080	0.062	0.024	0%	17%	41%	96%
14	5	Moderate	0.056	0.029	0.070	0.018	27%	78%	7%	97%
15	5	Strong	0.015	0.016	0.063	0.013	93%	92%	1%	97%
16	6	None	0.129	0.081	0.045	0.028	7%	39%	76%	96%
17	6	Moderate	0.141	0.092	0.021	0.020	0%	5%	95%	96%
18	6	Strong	0.127	0.085	0.015	0.015	0%	1%	97%	97%

The reference HR was derived by calculating the counterfactual outcome for each individual and estimating the hazard ratio in the resulting sample of 20,000 individuals. The mean across 500 simulations was then used as “unbiased” source. For scenarios where there is no treatment effect, the reference HR was set to 1. LOCF: last observation carried forward; RND: rounding; MLMI-RND: multilevel imputation with rounding; MLMI-PMM: multilevel imputation with predictive mean matching.

Finally, simulation study results in Supplemental Figures S5 and S6 indicate that estimated confidence intervals (CIs) were too narrow when adopting LOCF, rounding, AVG, or multilevel imputation with rounding. The poorest coverage rates were obtained for LOCF, which yielded 0% coverage in many scenarios. Coverage improved when adopting multilevel imputation, especially when draws for the EDSS scores derived using MLMI-PMM. Although the coverage for multilevel imputation was too low when generated draws were rounded to the nearest half-integer, MLMI-PMM yielded a coverage close to the nominal level of 95%.

## 5 Case study

We used data from the Multiple Sclerosis Partners Advancing Technology and Health Solutions (MS PATHS), a collaborative network of healthcare institutions collecting standardized measurements on patients living with MS.^28^ MS PATHS is the first demonstration project of a learning health system in MS, collecting standardized clinical and imaging data on MS patients across 10 healthcare institutions in the United States (*n* = 7) and in European Union (*n* = 3). Patients attend an initial visit upon enrollment and subsequent follow-up visits obeying no pre-specified schedule.

### 5.1 Cohort definition and methods

The study population consisted of new users of DMF or fingolimod (FTY) between November 2015 and July 2021 with any type of MS. New users were defined using the self-reported current DMT recorded at each visit. A treatment sequence consisted of consecutive visits on the same DMT. We assumed that the first visit of the treatment sequence corresponded to treatment initiation and labeled that first visit as a baseline. We defined the follow-up time from baseline until the last MS PATHS follow-up visit, treatment switch, or treatment discontinuation, whichever comes first. Disease progression was defined with the Patient Determined Disease Steps (PDDS) score, a patient-reported measure of disability strongly correlated with EDSS with ordinal scores ranging from 0 (normal) to 8 (bedridden) in 1-point increment.^29^ The outcome was time to CDP calculated as the time in days from baseline until disease progression, defined as a 1-point increase in PDDS score from baseline PDDS, provided the increase can be confirmed at a follow-up visit 3 months later. Patients with complete baseline data were kept for the primary analysis. Additional details on cohort definitions are available in A4.1 of the Supplemental Information.

We compared LOCF, rounding, and the proposed multi-level modeling approach to generate imputations for PDDS score and derive the corresponding time to CDP. We reconstructed the entire PDDS trajectories following pre-specified grids of visits every 3 and 6 months. If no CDP was identified, patients were censored at the end of the treatment sequence. If a disease progression was observed less than 3 months before the end of treatment sequence, we allowed to impute beyond the follow-up time to confirm the progression. An inverse probability weighted Cox regression model accounting for clustering by MS PATHS site was used to compare the effect of DMF vs FTY on time to CDP with marginal hazard ratios. The following baseline covariates were considered: age, sex, MS type, years of education, disease duration, PDDS score, self-reported number of relapses in the past 12 months, prior DMT classified as no prior DMT or high, medium, or low DMT efficacy,^b^ history of cardiovascular disease, and history of diabetes. Corrected sandwich variance estimation was used to derive robust SEs using stabilized weights.^27^ LOCF and rounding each generated single imputation for time to CDP, so the Cox model was applied once for both methods. With our multi-level modeling approach, we generated 100 imputations, estimated the HR and corresponding SE for each imputed time to CDP, and combined the results using Rubin's rule. Details on the multi-level model specification and weight calculation are available from A4.2 of the Supplemental Information.

As a sensitivity analysis, we adopted multiple imputations by chained equations in the entire cohort with incomplete baseline data. To this purpose, we implemented LOCF, rounding and the proposed multi-level modeling as a conditional modeling approach in the R package *mice*. Time-invariant variables were then imputed using PMM at level 2 using the function *mice.impute.2lonly.pmm*.

### 5.2 Results

As of July 2021, the MS PATHS database included 16,152 patients. Of those, we identified 254 and 202, new users of DMF and FTY, respectively, with complete baseline data. Table 3 shows the baseline characteristics of the cohort by treatment group.

**Table 3.** Baseline characteristics of 456 patients with complete data who initiated DMF or FTY in MS PATHS.

Characteristics	DMF (*n* = 254)	FTY (*n* = 202)
Age, mean (SD)	45 (10)	41 (10)
Male, *n* (%)	49 (19)	47 (23)
Years of education, mean (SD)	15 (3)	14 (3)
MS type, *n* (%)		
Relapsing MS (remitting/progressive)	199 (78)	175 (87)
Primary progressive MS	12 (5)	8 (4)
Secondary progressive MS	43 (17)	19 (9)
Number of relapses in the past 12 months, *n* (%)		
0	121 (48)	104 (51)
1	69 (27)	52 (26)
2	38 (15)	28 (14)
≥3	26 (10)	18 (9)
Prior DMT efficacy*, *n* (%)		
High	10 (4)	15 (7)
Medium	13 (5)	26 (13)
Low	105 (41)	77 (38)
None	126 (50)	84 (42)
History of cardiovascular disease, *n* (%)	93 (37)	63 (32)
History of diabetes, *n* (%)	21 (8)	11 (5)
PDDS score, *n* (%)		
0–1	160 (63)	152 (75)
2–3	51 (20)	36 (18)
4–5	31 (12)	8 (4)
≥6	12 (5)	6 (3)
Visit count, *n* (%)		
1	62 (24)	40 (20)
2	98 (39)	50 (25)
3	45 (18)	48 (24)
4	24 (9)	23 (11)
≥5	25 (10)	41 (20)

*Interferons and glatiramer acetate were classified as low efficacy. Teriflunomide, methotrexate and mycophenolate mofetil were classified as medium efficacy. Alemtuzumab, ocrelizumab, natalizumab, and rituximab were classified as high efficacy. DMF: dimethyl fumarate; DMT: disease-modifying therapy; FTY: fingolimod; MS: multiple sclerosis; PDDS: Patient-Determined Disease Steps; SD: standard deviation.

**Figure 3.**
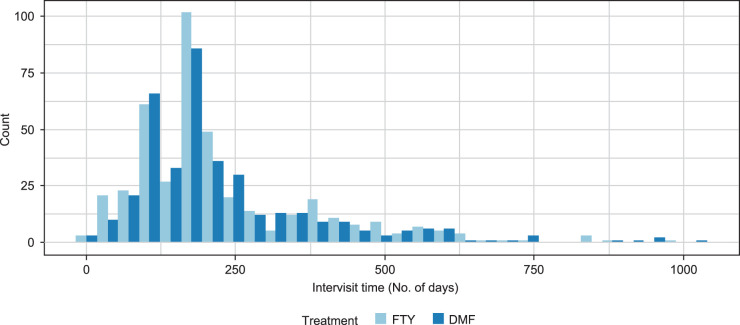
Distribution of number of days between consecutive visits for 192 new DMF users and 162 new FTY users having at least one follow-up visit beyond baseline. DMF: dimethyl fumarate; FTY: fingolimod.

The median number of visits per patient was 2 (DMF: 2, FTY: 3), with 102 patients (DMF: 62, FTY: 40) having only a baseline visit. The distribution of patient visits was similar for both treatment groups (Figure 3), and the median duration between consecutive visits was 185 days (interquartile range (IQR): 120; 261) for DMF, and 188 days (IQR: 120; 267.5) for FTY. Despite these similarities between the treatment groups, DMF and FTY patients substantially differed with respect to most baseline characteristics (A4.3 of the Supplemental Information). When adjusting for confounders, we found that DMF reduced PDDS by 0.02 point per year (not statistically significant) as compared to FTY, and that the residual autocorrelation between monthly PDDS scores was 0.13 (A4.4 of the Supplemental Information).

We subsequently imputed PDDS scores on a 3-month and 6-month grid using LOCF, rounding and the proposed multilevel model. Results in the Supplemental Information indicate that these methods sometimes lead to different disease trajectories (A4.5) and survival data (A4.6). For example, we found that the median censoring time was 270 days for LOCF, 231 days for rounding, and 222 days for multilevel imputation with PMM. We also found some differences in overall survival. For example, the one-year risk of developing CDP varied between 17% (LOCF and MLMI) and 21% (rounding) for patients that received FTY, and between 14% (LOCF and MLMI) and 23% (rounding) for patients that received DMF.

The estimated HR and corresponding 95% CI are shown in Table 4 for the three imputation methods using either a 3- or 6-month pre-specified grid of visits. There was no evidence of a relative difference in effectiveness between DMF and FTY for any imputation method. All methods yielded an HR close to 1, with LOCF providing the largest SEs. SEs generally increased when the treatment effect was evaluated on a 6-month (rather than 3-month) grid. Similar results were found when chained equations imputation was applied in the cohort of 601 patients with incomplete baseline data.

**Table 4.** Estimated HR of DMF vs FTY for time to CDP with three methods according to two imputation grids.

			3-month grid		6-month grid	
Method	N*	CCA	HR (95% CI)	SE of log(HR)	HR (95% CI)	SE of log(HR)
LOCF	354 (456)	Yes	0.96 (0.57; 1.62)	0.27	1.10 (0.63; 1.93)	0.29
RND	354 (456)	Yes	1.16 (0.88; 1.53)	0.14	1.13 (0.86; 1.49)	0.14
MLMI-RND	354 (456)	Yes	0.86 (0.68; 1.09)	0.12	0.86 (0.52; 1.42)	0.26
MLMI-PMM	354 (456)	Yes	0.89 (0.63; 1.26)	0.17	0.89 (0.56; 1.42)	0.24
LOCF	479 (601)	No	1.02 (0.75; 1.38)	0.16	1.12 (0.81; 1.55)	0.16
RND	479 (601)	No	1.08 (0.90; 1.29)	0.09	1.12 (0.91; 1.39)	0.11
MLMI-RND	479 (601)	No	0.91 (0.73; 1.13)	0.11	0.88 (0.64; 1.21)	0.16
MLMI-PMM	479 (601)	No	0.92 (0.74; 1.16)	0.12	0.87 (0.64; 1.19)	0.16

*N*: effective sample size (and total sample size); CCA: complete case analysis (if “no”, missing baseline data were imputed using multiple imputation); CDP: confirmed disease progression; CI: confidence interval; DMF: dimethyl fumarate; FTY: fingolimod; HR: hazard ratio; LOCF: last observation carried forward; MLMI-PMM: multilevel imputation with predictive mean matching; MLMI-RND: multilevel imputation with rounding; RND: rounding.

*Treatment effect estimates are only informed by patients with at least two visits. The effective sample size may therefore differ from the total number of patients (all of which are used to estimate the multilevel imputation model).

## 6 Discussion

In this paper, we evaluated various methods for imputing longitudinal observations at arbitrary measurement times and deriving survival outcomes for subsequent comparative effectiveness research. These methods can be used in datasets where repeated measurements are available for variables of interest to facilitate analyses that require observations at specific time points or intervals. We extended previously proposed imputation methods for multicenter data,^21^ and adopted additional random effect terms to account for the clustering of repeated observations. Further, we incorporated an autocorrelation structure to exploit the potential presence of correlated residual errors. This situation typically arises when the imputation model does not adequately capture the continuous time process of the repeated measurements; for instance, because the frequency of visits is driven by unobserved information. Moreover, to facilitate the imputation of semi-continuous and skewed variables, we implemented PMM. Finally, we coupled the proposed methods with a comparative effectiveness analysis by deriving a survival outcome from the imputed longitudinal trajectories and using inverse probability of treatment weighting to account for confounding. The proposed imputation approach can directly be implemented in MICE, and is, therefore, suitable to handle multiple and mixed-type time-dependent covariates that typically arise in electronic healthcare registries.

Results from an extensive simulation study demonstrated that our proposed method yields more accurate imputations than commonly used methods based on time series analysis. When the imputed values were used for statistical inference, we found that hierarchical imputation was least prone to bias and attained coverage levels that were close to the nominal level. Although the proposed imputation methods are designed for situations where observations are missing at random, results from the simulation study suggested that they may also work reasonably well when data are MNAR. Most likely, imputation performance under MNAR conditions strongly depends on the presence of autocorrelation between successive observations, as this allows to reduce bias in the imputed values. Further research is needed to evaluate this issue. Further, we found that MLMI offers better statistical properties when based on PMM. Possibly, this is because the variation of imputed values decreases when they are simply rounded to the nearest-half integer. Conversely, PMM generates imputed values by resampling from the observed data and therefore offers improved capabilities of preserving uncertainty.

The simulation study setup was motivated by the MS clinical context which uses the time to CDP derived from longitudinal measurements of the EDSS score. Thus, we demonstrated the performance of the new method for a particular application, that is, to recover a time-to-event outcome from a longitudinal variable captured at irregular measurement points within and across individuals. Other disease areas beyond MS use similar endpoints defined as the time from baseline to the occurrence of an event, where the event is derived from an underlying continuous or semi-continuous process (e.g., a biomarker or a score on a scale) only measured at certain time points. Nevertheless, the proposed method is suitable in much broader contexts, with any longitudinal outcome or variable. Moreover, the MS clinical context also informed the intermittent visit patterns in the simulation study, which were chosen to reflect realistic covariate- and outcome-driven follow-up patterns in MS. This led to a 1–2 time-points per year for collecting the longitudinal variable of interest. Other applications may have more or less frequent visits. For example, it would be more realistic to consider set-ups with more frequent visits for diabetes patients needing to monitor hemoglobin A1C every 3 months.

In the illustrative example, we used the proposed imputation method to recover the trajectory of PDDS scores in MS patients identified in MS PATHS, an ongoing demonstration project of a learning health system collecting data in clinical practice. The proposed method imputed PDDS scores according to a standardized follow-up visit schedule, which allowed one to observe and confirm a disability progression. We subsequently derived time to CDP, an important clinical outcome in MS, and evaluated the comparative effectiveness between DMF and FTY in terms of CDP. By adopting hierarchical imputation, we were able to obtain smaller SEs for treatment effect estimates (as compared to LOCF) and to include patients with missing covariate data in the analysis. All imputation methods suggested similar effectiveness of DMF and FTY, substantiating evidence from the literature.^30,31^

Some limitations need to be considered in this research. First, we considered that a reasonable number of repeated observations are available to generate imputations. When longitudinal responses are very sparse or limited in number, regression models with autocorrelation may not well be capable to accurately recover their temporal relationship. As MS studies using RWD typically have shorter follow-up times than the one used in the simulation study and may thus have less repeated measurements, it would be worth evaluating the methods in this context. Second, we implemented the R package *nlme* to estimate and propagate uncertainty for parameters of the hierarchical imputation model. This package uses a nonlinear optimization function and sometimes leads to non-positive definite approximate variance-covariance matrices. The generation of random parameter draws may therefore suffer when datasets are relatively small or do not contain many repeated observations. Third, we did not evaluate adjustment for flexible covariate effects during imputation. It may, for instance, be desirable to include time-varying or non-linear covariate effects in the imputation model when explanatory variables also vary over time. When advanced covariate adjustments are not feasible during imputation (e.g., due to limited sample size), implementation of an autocorrelation structure may help to reduce bias. Fourth, results from the simulation study indicate that hierarchical imputation sometimes yields SEs that are too large or too small. Possibly, this is because the simulation study involves a discrete outcome that cannot be described using common statistical distributions. Although we adopted PMM to address this issue, its implementation is no panacea and may still be prone to some amount of bias. Finally, we focused on the imputation of longitudinal variables that represent a study outcome to facilitate the recovery of study endpoints. Hierarchical imputation of longitudinal variables can, however, also be used to restore missing baseline characteristics or to reconstruct entire exposure trajectories.

In summary, we recommend MLMI to address the presence of missing values in registry data with repeated measurements. In contrast to ad-hoc solutions that are based on time series analysis, our approach leads to less bias and is better capable of preserving adequate levels of uncertainty.

## Supplemental Material

sj-docx-1-smm-10.1177_09622802231172032 - Supplemental material for Methods for comparative effectiveness based on time to confirmed disability progression with irregular observations in multiple sclerosis Click here for additional data file. Supplemental material, sj-docx-1-smm-10.1177_09622802231172032 for Methods for comparative effectiveness based on time to confirmed disability progression with irregular observations in multiple sclerosis by Thomas PA Debray, Gabrielle Simoneau, Massimiliano Copetti, Robert W Platt, Changyu Shen, Fabio Pellegrini and Carl de Moor in Statistical Methods in Medical Research

sj-zip-2-smm-10.1177_09622802231172032 - Supplemental material for Methods for comparative effectiveness based on time to confirmed disability progression with irregular observations in multiple sclerosis Click here for additional data file. Supplemental material, sj-zip-2-smm-10.1177_09622802231172032 for Methods for comparative effectiveness based on time to confirmed disability progression with irregular observations in multiple sclerosis by Thomas PA Debray, Gabrielle Simoneau, Massimiliano Copetti, Robert W Platt, Changyu Shen, Fabio Pellegrini and Carl de Moor in Statistical Methods in Medical Research

## Notes

a. Clinical trials in MS often include an additional visit 3 months after the last planned follow-up visit to confirm a progression if disease progression was observed at the last visit.

b. Interferons and glatiramer acetate were classified as low efficacy. Teriflunomide, methotrexate and mycophenolate mofetil were classified as medium efficacy. Alemtuzumab, ocrelizumab, natalizumab and rituximab were classified as high efficacy.

## References

[1] Sarri G Bennett D Debray T , et al. ISPE-endorsed guidance in using electronic health records for comparative effectiveness research in COVID-19: opportunities and trade-offs. Clin Pharma Therapeut 2022; 112: 990–999. doi: 10.1002/cpt.2560. PMC9087010 35170021

[2] Agniel D Kohane IS Weber GM . Biases in electronic health record data due to processes within the healthcare system: retrospective observational study. Br Med J 2018; 361: k1479. 29712648 10.1136/bmj.k1479 PMC5925441

[3] Cook JA Collins GS . The rise of big clinical databases. Br J Surg 2015; 102: e93–101. 25627139 10.1002/bjs.9723

[4] Makady A Stegenga H Ciaglia A , et al. Practical implications of using real-world evidence (RWE) in comparative effectiveness research: learnings from IMI-GetReal. J Comp Eff Res 2017; 6: 485–490. 28857631 10.2217/cer-2017-0044

[5] Riester K Kappos L Selmaj K , et al. Impact of informative censoring on the treatment effect estimate of disability worsening in multiple sclerosis clinical trials. Mult Scler Relat Disord 2020; 39: 101865. 31835206 10.1016/j.msard.2019.101865

[6] Woolley SB Cardoni AA Goethe JW . Last-observation-carried-forward imputation method in clinical efficacy trials: review of 352 antidepressant studies. Pharmacotherapy 2009; 29: 1408–1416. 19947800 10.1592/phco.29.12.1408

[7] Lachin JM . Fallacies of last observation carried forward analyses. Clin Trials 2016; 13: 161–168. 26400875 10.1177/1740774515602688 PMC4785044

[8] Siddiqui O Hung HMJ O’Neill R . MMRM Vs. LOCF: a comprehensive comparison based on simulation study and 25 NDA datasets. J Biopharm Stat 2009; 19: 227–246. 19212876 10.1080/10543400802609797

[9] Huque MH Carlin JB Simpson JA , et al. A comparison of multiple imputation methods for missing data in longitudinal studies. BMC Med Res Methodol 2018; 18: 168. 30541455 10.1186/s12874-018-0615-6 PMC6292063

[10] Bartlett JW Seaman SR White IR , et al. Multiple imputation of covariates by fully conditional specification: accommodating the substantive model. Stat Methods Med Res 2015; 24: 462–487. 24525487 10.1177/0962280214521348 PMC4513015

[11] Audigier V White IR Jolani S , et al. Multiple imputation for multilevel data with continuous and binary variables. Stat Sci 2018; 33: 160–183.

[12] Enders CK Mistler SA Keller BT . Multilevel multiple imputation: a review and evaluation of joint modeling and chained equations imputation. Psychol Methods 2016; 21: 222–240. 26690775 10.1037/met0000063

[13] Grund S Lüdtke O Robitzsch A . Multiple imputation of missing data for multilevel models: simulations and recommendations. Organ Res Methods 2018; 21: 111–149.

[14] Wijesuriya R Moreno-Betancur M Carlin JB , et al. Evaluation of approaches for multiple imputation of three-level data. BMC Med Res Methodol 2020; 20: 207. 32787781 10.1186/s12874-020-01079-8 PMC7422505

[15] Goldstein BA Navar AM Pencina MJ , et al. Opportunities and challenges in developing risk prediction models with electronic health records data: a systematic review. J Am Med Inform Assoc 2017; 24: 198–208. 27189013 10.1093/jamia/ocw042 PMC5201180

[16] Curran PJ Obeidat K Losardo D . Twelve frequently asked questions about growth curve modeling. J Cogn Dev 2010; 11: 121–136. 21743795 10.1080/15248371003699969 PMC3131138

[17] Herle M Micali N Abdulkadir M , et al. Identifying typical trajectories in longitudinal data: modelling strategies and interpretations. Eur J Epidemiol 2020; 5: 205–222. 10.1007/s10654-020-00615-6 PMC7154024 32140937

[18] Erler NS Rizopoulos D Jaddoe VW , et al. Bayesian Imputation of time-varying covariates in linear mixed models. Stat Methods Med Res 2019; 28: 555–568. 29069967 10.1177/0962280217730851 PMC6344996

[19] Anderson RL . The problem of autocorrelation in regression analysis. J Am Stat Assoc 1954; 49: 113–129.

[20] Pinheiro JC Bates DM . Mixed-effects models in S and S-PLUS. New York, NY [u.a.]: Springer, 2000. (Statistics and Computing).

[21] Jolani S . Hierarchical imputation of systematically and sporadically missing data: an approximate Bayesian approach using chained equations. Biom J 2018; 60: 333–351. 28990686 10.1002/bimj.201600220

[22] Resche-Rigon M White IR . Multiple imputation by chained equations for systematically and sporadically missing multilevel data. Stat Methods Med Res 2016; 27: 1634–1649. 27647809 10.1177/0962280216666564 PMC5496677

[23] Rubin DB . Multiple imputation for nonresponse in surveys. New York: Wiley, 1987.

[24] Vink G Frank LE Pannekoek J , et al. Predictive mean matching imputation of semicontinuous variables: PMM imputation of semicontinuous variables. Stat Neerl 2014; 68: 61–90.

[25] Kappos L Butzkueven H Wiendl H , et al. Greater sensitivity to multiple sclerosis disability worsening and progression events using a roving versus a fixed reference value in a prospective cohort study. Mult Scler 2018; 24: 963–973. 28554238 10.1177/1352458517709619 PMC6029149

[26] Brown MG Asbridge M Hicks V , et al. Estimating typical multiple sclerosis disability progression speed from clinical observations. Bayer A, editor. PLoS ONE 2014; 9: e105123. 25329469 10.1371/journal.pone.0105123 PMC4201451

[27] Shu D Young JG Toh S , et al. Variance estimation in inverse probability weighted Cox models. Biometrics 2020; 77: 1101–1117. 32662087 10.1111/biom.13332 PMC12534113

[28] Mowry EM Bermel RA Williams JR , et al. Harnessing real-world data to inform decision-making: multiple sclerosis partners advancing technology and health solutions (MS PATHS). Front Neurol 2020; 11: 632. 32849170 10.3389/fneur.2020.00632 PMC7426489

[29] Learmonth YC Motl RW Sandroff BM , et al. Validation of patient determined disease steps (PDDS) scale scores in persons with multiple sclerosis. BMC Neurol 2013; 13: 37. 23617555 10.1186/1471-2377-13-37 PMC3651716

[30] Vollmer B Ontaneda D Bandyopadhyay A , et al. Discontinuation and comparative effectiveness of dimethyl fumarate and fingolimod in 2 centers. Neurol Clin Pract 2018; 8: 292–301. 30140580 10.1212/CPJ.0000000000000487 PMC6105060

[31] Braune S Grimm S van Hövell P , et al. Comparative effectiveness of delayed-release dimethyl fumarate versus interferon, glatiramer acetate, teriflunomide, or fingolimod: results from the German NeuroTransData registry. J Neurol 2018; 265: 2980–2992. 30327931 10.1007/s00415-018-9083-5 PMC6244642

