# Supplementary material for: Methods for comparative effectiveness based on time to confirmed disability progression with irregular observations in multiple sclerosis
Source: Stat Methods Med Res. 2023 Jun 11;32(7):1284–99. doi: 10.1177/09622802231172032 (PMC10500950; doi:10.1177/09622802231172032)
Supplement: sj-docx-1-smm-10.1177_09622802231172032 - Supplemental material for Methods for comparative effectiveness based on time to confirmed disability progression with irregular observations in multiple sclerosis [file sj-docx-1-smm-10.1177_09622802231172032.docx]

Supporting Information for “Multiple imputation using chained equations for clustered longitudinal data: A simulation study and a case study in multiple sclerosis”

# A1 | NOTATION

| **Symbol** | **Description** |
| --- | --- |
| $i$ | An indicator variable to denote the patient |
| $j$ | An indicator variable to denote the center |
| $v$ | An indicator variable to denote the visit, with $v=1,\ldots,n_{ij}$ |
| $x_{ij}$ | A binary variable indicating the received treatment at the first patient visit ($x_{ij}=0$ for the control treatment, and $x_{ij}=1$ for the active treatment). |
| $z_{mij}$ | Observed value for covariate *m* at the first patient visit |
| $n_{ij}^{\text{obs}}$ | A count variable representing the total number of visits for patient $i$ in center $j$ |
| $n_{ij}^{\text{mis}}$ | A count variable indicating the total number of visit times for which the outcomes are unavailable and for which an imputation would be desired. |
| $\boldsymbol{y}_{\boldsymbol{vij}}^{\text{obs}}$ | A continuous variable denoting the observed outcome at visit *v* for patient $i$ in center $j$. The outcome at the start of treatment is given as $\boldsymbol{y}_{\boldsymbol{1}\boldsymbol{ij}}^{\text{obs}}$. |
| $\boldsymbol{y}_{\boldsymbol{ij}}^{\text{mis}}$ | A $n_{ij}^{\text{mis}}\times1$ vector containing the missing outcomes at visit times $\boldsymbol{t}_{\boldsymbol{ij}}^{\text{mis}}$ |
| $\boldsymbol{y}_{\boldsymbol{ij}}^{\text{*,mis}}$ | A $n_{ij}^{\text{mis}}\times1$ vector containing a random draw for the missing outcomes at visit times $\boldsymbol{\Phi}_{\boldsymbol{ij}}$ |
| $t_{vij}^{\text{obs}}$ | A continuous variable denoting the visit time. By definition, $t_{vij}^{\text{obs}}\geq0$ with $t_{1ij}^{\text{obs}}=0$. |
| $\boldsymbol{t}_{\boldsymbol{ij}}^{\text{mis}}$ | An $n_{ij}^{\text{mis}}\times1$ vector indicating the visit times for which outcome imputations are needed |
| $\boldsymbol{1}_{p}$ | A $p\times1$ vector of ones |

Table 1 Notation in the main manuscript

# A2 | SIMULATION STUDY

## A2.1 | Details of the data-generating mechanism

For each individual *i*=1, ..., 10000, we generated disease trajectories over 60 months. Individual disease trajectories were characterized by a continuous score $m_{ti}$ assessed at discrete time points $t=0, 1,\ldots, 60$ months. The continuous score $m_{ti}$ can be thought as a continuous and unbounded EDSS score, which facilitated the implementation of the data-generating mechanism. The vector of 61 hypothetical EDSS scores is affected by a lag-1 temporal autocorrelation. The magnitude for the autocorrelation between successive months was derived from estimates from a randomized trial evaluating the efficacy of DMT in patients with relapsing–remitting MS, and set to 0.8 (1). As a final step, the random draws for $m_{ti}$ are rounded to the nearest half-integer and truncated between 0 and 9.5 to obtain the observable EDSS scores $y_{ti}$.

We generated two individual-level characteristics: a random effect $a_{i}\sim\text{Normal}(\mu=0, \sigma=1.46)$ to personalize disease severity at treatment start (baseline) and $\text{age}_{i}$, a covariate to define age at treatment start. The distribution of $\text{age}_{i}$ (Figure 1) was chosen to mimic the distribution of age in the case study as

$$\text{age}_{i}\sim\text{truncated Normal}(\mu=42.41, \sigma=10.53, a=18, b=\text{Inf}).$$

Individuals received DMT A ($x_{i}=0$) or DMT B ($x_{i}=1$) at time $t=0$ months according to $\text{age}_{i}$ (Figure ) as

$$x_{i}\sim\text{Bernoulli}\left( p=\text{logit}^{-1}\left( 0.7-0.032 \text{age}_{i}-0.0001 \text{age}_{i}^{2} \right) \right).$$

To allow for heterogeneity in baseline continuous EDSS scores between centers, we introduced another random effect $b_{\text{center}_{i}}\sim\text{Normal}(\mu=0, \sigma=0.20)$. The continuous scores $m_{ti}$ are generated according to:

$$\left[ \begin{matrix} m_{0i} \\ m_{1i} \\ \vdots\\ m_{60i} \end{matrix} \right]=1.3295+a_{i}+b_{\text{center}_{i}}+0.014\left[ \begin{matrix} t=0 \\ t=1 \\ \vdots\\ t=60 \end{matrix} \right]+\delta x_{i}\left[ \begin{matrix} t=0 \\ t=1 \\ \vdots\\ t=60 \end{matrix} \right]+0.05 \text{age}_{i}+\left[ \begin{matrix} \epsilon_{0i} \\ \epsilon_{1i} \\ \vdots\\ \epsilon_{60i} \end{matrix} \right]$$

The model for $m_{ti}$ assumed an AR1 structure for the continuous EDSS scores of a given individual over time through the distribution of errors:

$$\left[ \begin{matrix} \epsilon_{0i} \\ \epsilon_{1i} \\ \vdots\\ \epsilon_{60i} \end{matrix} \right]\sim N\left( 0,\left[ \begin{matrix} {0.5}^{2} & {0.8}^{1}{0.5}^{2} & \ldots& {0.8}^{60}{0.5}^{2} \\ {0.8}^{1}{0.5}^{2} & {0.5}^{2} & \ldots& {0.8}^{59}{0.5}^{2} \\ \vdots& & \ddots& \vdots\\ {0.8}^{60}{0.5}^{2} & \ldots& \ldots& {0.5}^{2} \end{matrix} \right] \right)$$

Note that this structure is acceptable because the time between EDSS measurements were equally spaced in time (one month). The coefficient for the time of measurements *t* was chosen to reflect an annual EDSS increase of 0.168. The coefficient for the interaction between time and treatment $\delta$ was chosen according to the three types of treatment effects: 0 (treatment does not affect disease progression), -0.007 (moderate treatment effect for DMT B that accumulated over time), and -0.014 (strong treatment for DMT B which accumulated over time). The intercept and coefficient of age in the disease trajectory model were chosen to yield an average baseline continuous EDSS score of 3.45 (for an *average* center) given by:

$$E\left( m_{0} \right)=\mu_{0}=1.3295+0.05\times42.41=3.45$$

The standard deviation of the baseline continuous EDSS score (for an *average* center) is given by:

$${(\sigma_{0})}_{j}=\sqrt{{1.46}^{2}+{0.05}^{2}\times{10.53}^{2}+{0.5}^{2}}=1.63$$

When combining the 20 centers, the baseline continuous EDSS scores follow a mixture of Normal distributions further characterized by the variance of $b_{\text{center}_{i}}$, with standard deviation of

$$\begin{matrix} SD\left( m_{0} \right) & = & \sqrt{\sum_{j=1}^{20} \frac{1}{20}\left( {(\sigma_{0})}_{j}^{2}+{(\mu_{0})}_{j}^{2}-\mu_{0}^{2} \right)} \\ & = & \sqrt{{(\sigma_{0})}_{j}^{2}+\mu_{0}^{2}+\text{Var}\left( \left( \mu_{0} \right)_{j} \right)-\mu_{0}^{2}} \\ & = & \sqrt{\left( \sigma_{0} \right)_{j}^{2}+\text{Var}\left( \left( \mu_{0} \right)_{j} \right)} \end{matrix}$$

We thus have $SD\left( m_{0} \right)=\sqrt{{1.63}^{2}+{0.20}^{2}}=1.64$.

As a final step, the random draws $m_{ti}$ were rounded to the nearest half-integer and truncated between 0 and 9.5 to obtain the observable EDSS scores $y_{ti}$.


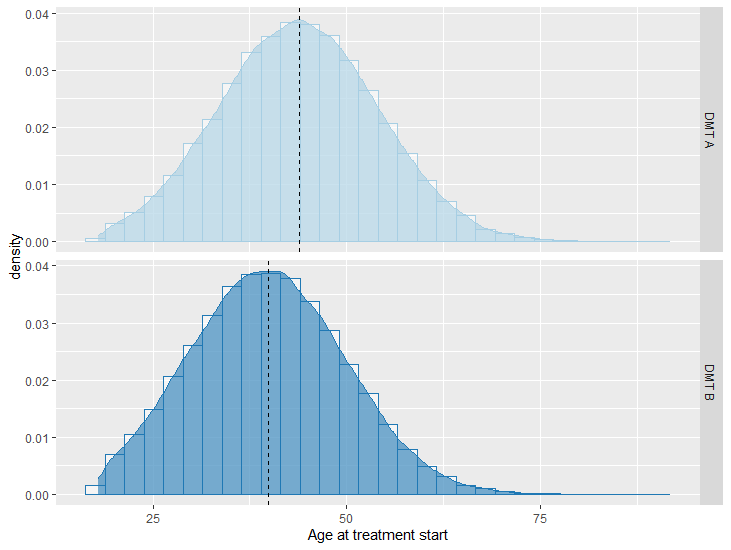


Figure 1 Distribution of baseline age in the simulation study by treatment group.

## A2.2 | Visit patterns

We generated irregular visit patterns by randomly setting generated EDSS scores $y_{ti}$ as missing for $t>0$ (the baseline EDSS score was always observed). In particular, the probability that $y_{ti}$ was observed at discrete time *t* is denoted by $Pr(o_{ti}=1)$ and defined by the following six strategies. Figure illustrate the distribution of $\Pr\left( o_{ti}=1 \right)$ over time for each strategy.

### **Visit pattern 1**: intermittent visits depend on center

The probability of observing $y_{ti}$ depended on the center *j* of individual *i* ($j=\text{center}_{i}$) according to

$$\Pr\left( o_{ti}=1 \right)=\text{logit}^{-1}(-1.94+u_{j})$$

with

$$u_{j}\sim N\left( \mu=0,\sigma=0.15 \right).$$

This missingness model implied that, for an *average* center, the probability of an individual’s visit at a discrete time $t>0$ is 12.6%. This led, on average, to a total of $1+60\times\text{expit}\left( -1.94 \right)=8.5$ visits per individual.

### **Visit pattern 2**: intermittent visits depend on baseline EDSS score

The probability of observing $y_{ti}$ depended on the EDSS score at baseline according to

$$\Pr\left( o_{ti}=1 \right)=\text{logit}^{-1}(-1.25-log(y_{0ij}))$$

This missingness model implied that, for an *average* center, the probability of an individual’s visit at a discrete time $t>0$ is $\text{expit}\left( -1.25-\log\left( 3.45 \right) \right)=7.6\%$. Note that, due to confounding, the average visit probability varied between DMT A and DMT B. In 500 simulated datasets, we found that this strategy led, on average, to a total of 7.8 visits per individual on DMT A and 8.5 visits per individual on DMT B.

### **Visit pattern 3**: intermittent visits depend on time and treatment

The probability of observing $y_{ti}$ depended on the time *t* and on the received treatment. This deletion mechanism represented situations where two treatments required different routine follow-up visit schedules. For individuals receiving DMT A, the probability of observing $y_{ti}$was 85% for $t=6, 12,\ldots, 60$ while it was set to 3% for all other time points. For individuals receiving DMT B, the probability of observing $y_{ti}$ was 67% for time points $t=9, 18,\ldots, 54$ and it was 3% otherwise. Visit pattern 3 reflected a situation where individuals treated with DMT A required more frequent visits, for example, to monitor side effects or complications.

### **Visit pattern 4**: intermittent visits depend on time and treatment

As visit pattern 3, this strategy also represented situations where two treatments require different routine follow-up visit schedules, but with DMT A requiring even more frequent visits than in the previous visit pattern. For individuals receiving DMT A, the probability of observing $y_{ti}$ was 35% for $t=3, 6,\ldots, 60$ while it was set to 3% for all other time points. For individuals receiving DMT B, the probability of observing $y_{ti}$ was 55% for time points $t=9, 18,\ldots, 54$ and it was 3% otherwise.

### **Visit pattern 5**: intermittent visits depend on center and treatment

The probability of observing $y_{ti}$ was given by

$$\Pr\left( o_{ti}=1 \right)=\text{logit}^{-1}(-1.6+u_{j}-0.7x_{i})$$

with

$$u_{j}\sim N(\mu=0,\sigma=0.15)$$

This missingness model implied that, for an *average* individual receiving DMT A, the visit probability at discrete time $t>0$ was $\text{expit}\left( -1.6 \right)=17\%$. The expected total number of visit for an individual receiving DMT A was therefore $1+60\times\text{expit}\left( -1.6 \right)=11$.

### **Visit pattern 6**: intermittent visits depend on treatment and current EDSS score

The probability of observing $y_{ti}$ depended on the received treatment and the EDSS score $y_{ti}$ at time *t* according to:

$$\Pr\left( o_{ti}=1 \right)=\text{logit}^{-1}(-0.5-0.5y_{ti}-0.5x_{i})$$

In 100 simulated datasets, we found that this strategy led, on average, to a total of 6.9 visits per individual on DMT A and 10.6 visits per individual on DMT B when there was no treatment effect. For a treatment effect of -0.007 and -0.014, the visit counts for DMT B increased to 11.3 and, respectively, to 12.2.


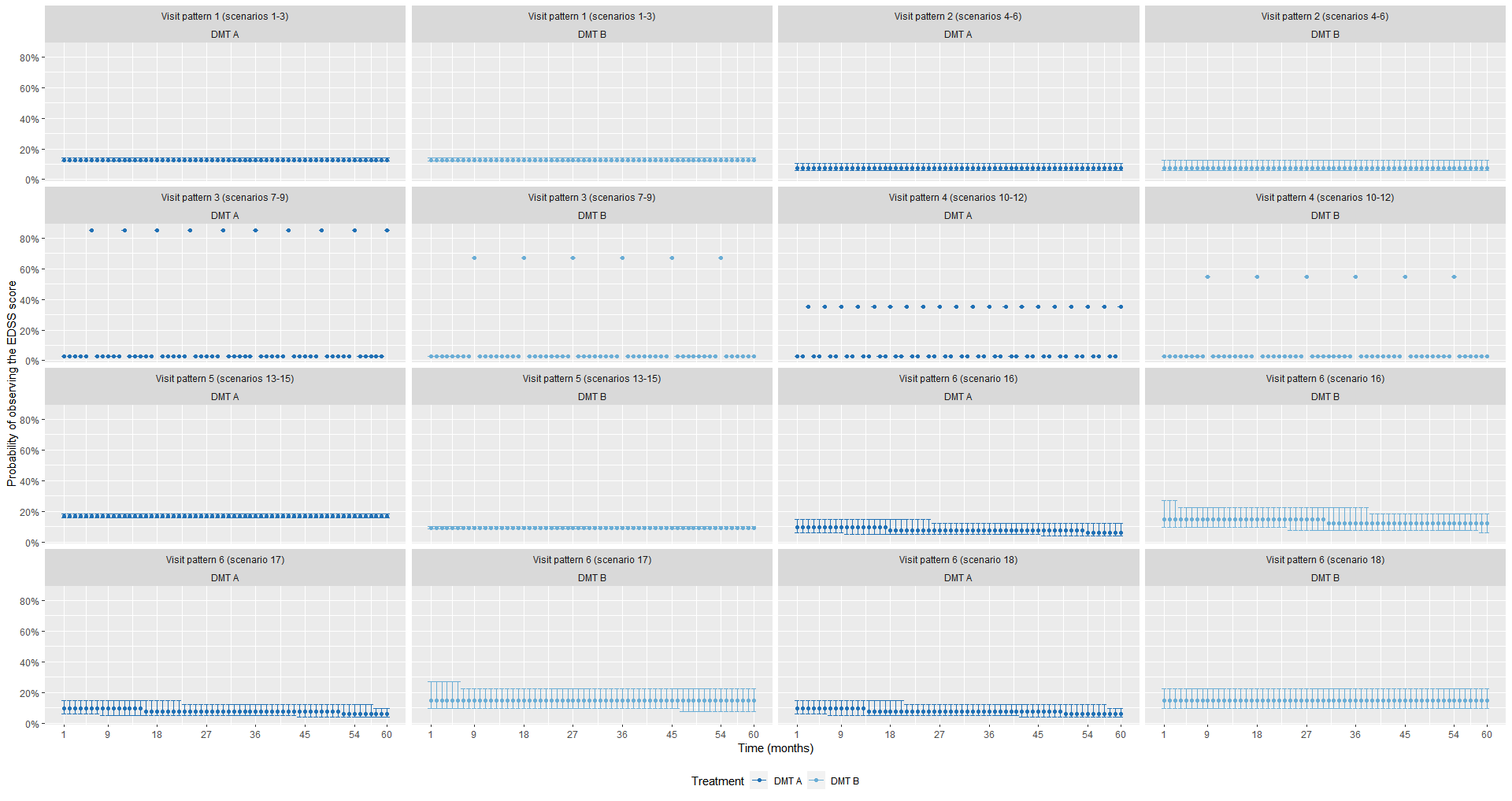


Figure 2 Visit probability over 60 months by treatment group. For each time point, the median and interquartile range of $\Pr\left( o_{ti}=1 \right)$, the probability of observing $y_{ti}$, is provided. For all scenarios, $\Pr\left( o_{0}=1 \right)=100\%$.

# A3 | SIMULATION RESULTS

## A3.1 | Root mean squared error of imputed EDSS scores

The root mean squared error (RMSE) of the imputed EDSS scores was derived within the observed follow-up at 3, 6, 12, …, up to a maximum of 60 months. Figure shows the distribution of the RMSE of imputed EDSS scores across 500 simulations for the four methods (last observation carried forward, rounding, multilevel modelling with rounded EDSS scores, multilevel modelling with predictive mean matching) across the types of treatment effects (none, moderate, strong). For multilevel multiple imputation, the RMSE was derived using the conditional mean of each missing EDSS score.


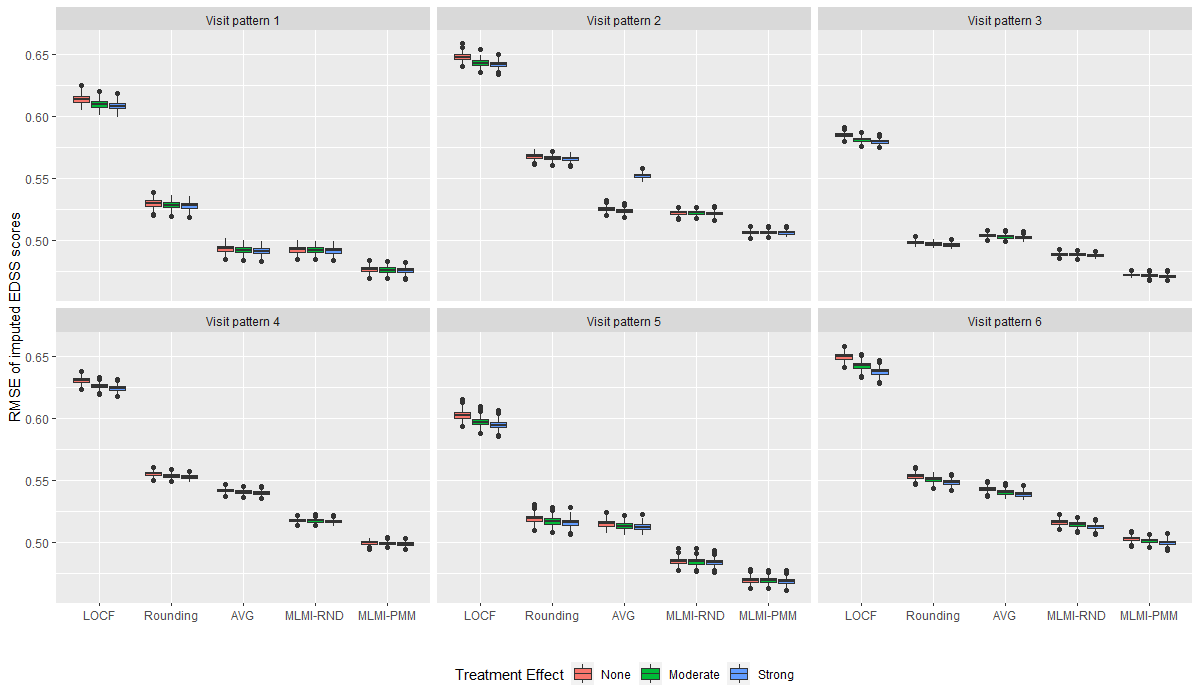


Figure 3 Root mean squared error (RMSE) of imputed EDSS scores. LOCF = last observation carried forward; AVG = average value imputation of the closest visit before and after the missing visit; MLMI-RND = multilevel multiple imputation with rounded EDSS scores; MLMI-PMM = multilevel multiple imputation with predictive mean matching.

## A3.2 | Estimated hazard ratios for the treatment effect


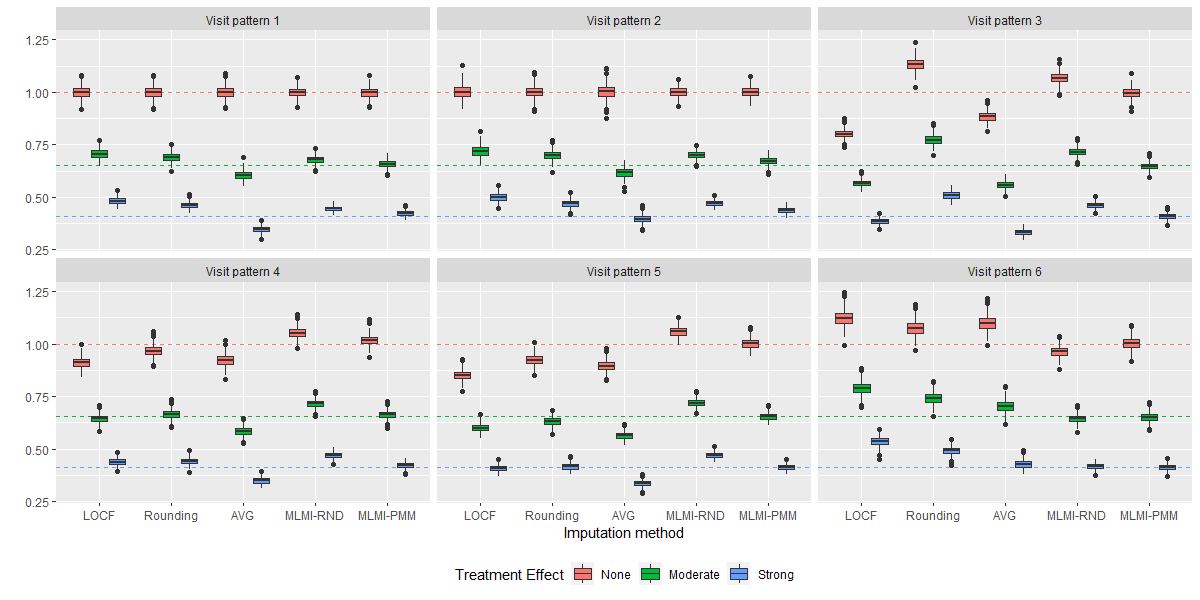


Figure 4 Estimated hazard ratios. LOCF = last observation carried forward; AVG = average value imputation of the closest visit before and after the missing visit; MLMI-RND = multilevel multiple imputation with rounded EDSS scores; MLMI-PMM = multilevel multiple imputation with predictive mean matching. The horizontal dashed lines represent the true hazard ratio.

## A3.3 | Coverage of the estimated treatment effect


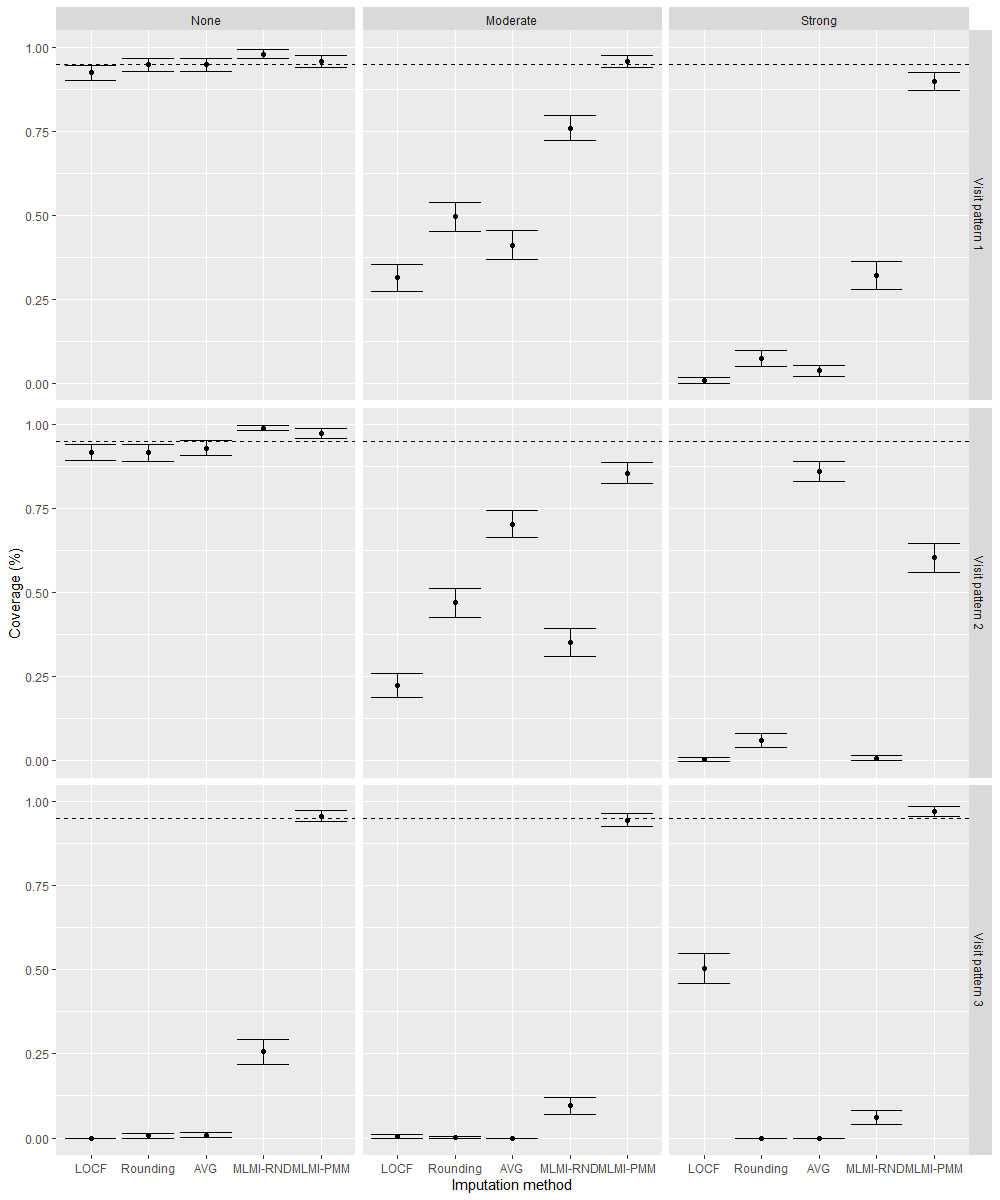


Figure 5 Coverage of the 95% confidence interval for the estimated treatment effect (“none”, “moderate” or “strong”). LOCF = last observation carried forward; AVG = average value imputation of the closest visit before and after the missing visit; MLMI-RND = multilevel multiple imputation with rounded EDSS scores; MLMI-PMM = multilevel multiple imputation with predictive mean matching.


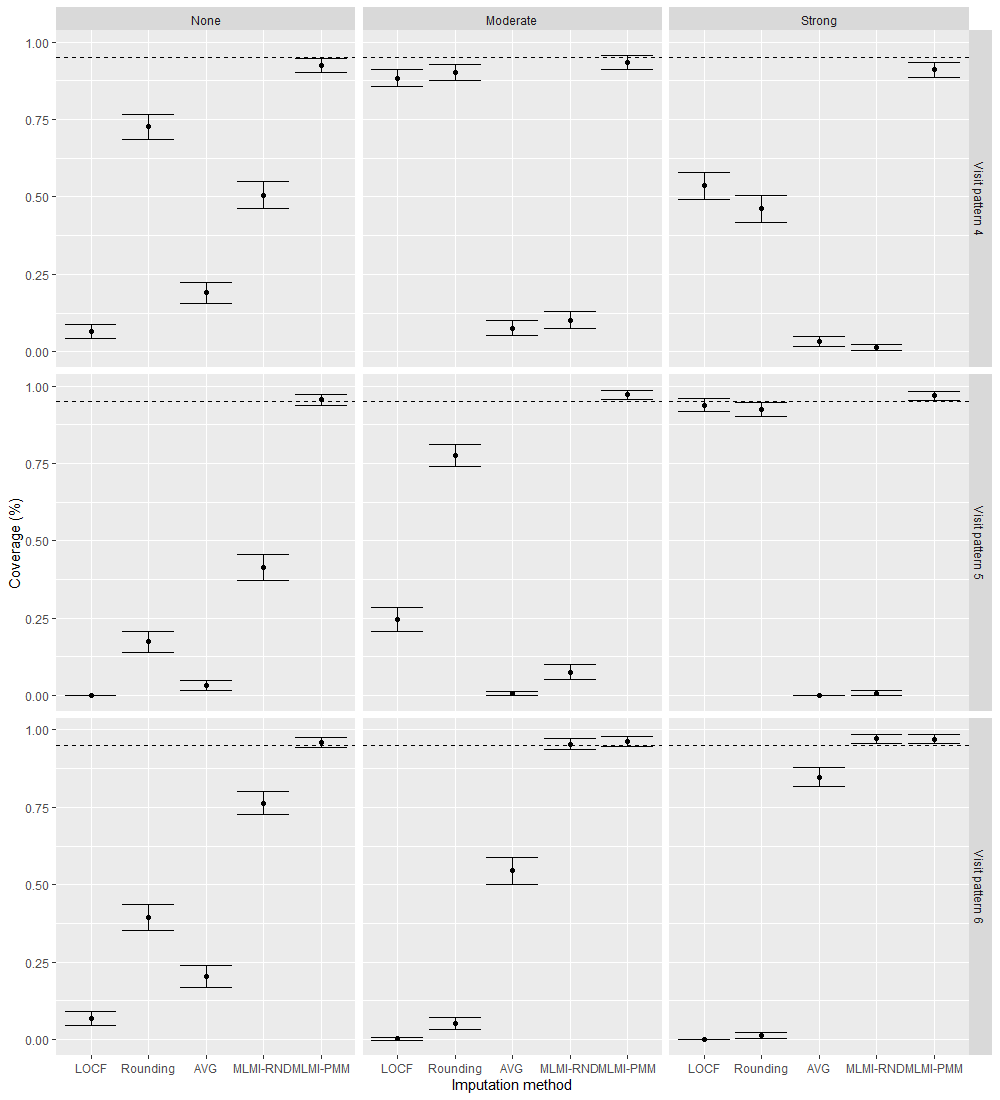


Figure 6 Coverage of the 95% confidence interval for the estimated treatment effect (“none”, “moderate” or “strong”). LOCF = last observation carried forward; AVG = average value imputation of the closest visit before and after the missing visit; MLMI-RND = multilevel multiple imputation with rounded EDSS scores; MLMI-PMM = multilevel multiple imputation with predictive mean matching.

# A4 | CASE STUDY

## A4.1 | Cohort construction

We selected patients from MS PATHS that received DMF or FTY and constructed their treatment sequences by identifying consecutive visits on the same DMT (Figure ). We focused on treatment sequences that started at a follow-up visit (not at the initial MS PATHS visit) and assumed that the baseline visit corresponded to treatment initiation. Treatment sequences that started at the initial MS PATHS visit were excluded because (1) the patient could have been treated with the self-reported DMT before enrolling in MS PATHS, and (2) pre-baseline patient characteristics were not available.


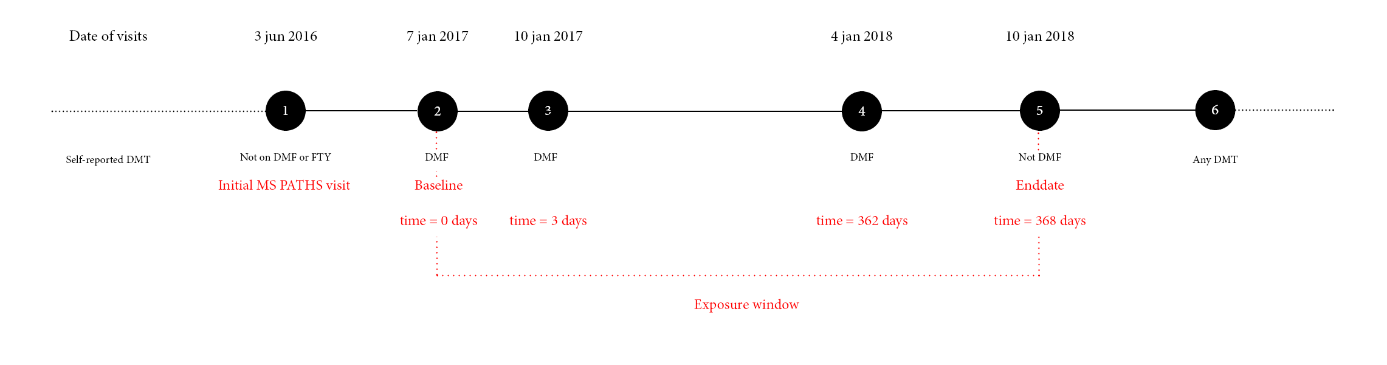


Figure 7 Construction of treatment sequences in MS PATHS for an example individual with 6 visits (black circles).

We distinguished between the two situations to establish the end date of a treatment trajectory.

1. If the current treatment sequence is followed by another sequence, the end date is set to the first visit date of the next treatment sequence.
2. If the current treatment sequence is the last one, the end date is set to the last visit date.

For each visit within a treatment sequence, we record the following information:

- Age at baseline
- Sex at baseline
- Years of education at baseline
- Disease duration at baseline
- Number of relapses in the 12 months before baseline
- MS type at baseline
- Prior DMT efficacy at baseline
- History of cardiovascular disease at baseline
- History of diabetes at baseline
- Site
- Current Patient Determined Disease Steps (PDDS) score

Baseline PDDS and number of relapses in the past 12 months were measured at the visit before the first visit of the treatment sequence to ensure that both variables were not affected by the studied treatment, which could have been initiated before the first visit of the sequence. For each individual, we selected the treatment sequence with the longest exposure window under DMF or FTY. In the resulting cohort, we removed individuals with missing MS type, or with MS type equal to “clinically isolated syndrome (CIS)” or “no CIS or MS diagnosis”.

## A4.2 | Specification of the imputation model

The imputation model for PDDS at visit *t* was specified as a linear combination of main effects for all covariates listed above, plus a time variable and an interaction between time and treatment. We used an spatial exponential correlation structure, accounting for the fact that individuals are nested within sites.

| **Variable** | **Explanation** |
| --- | --- |
| pdds | The PDDS score at time *time* |
| time | Number of days elapsed since baseline |
| time_x | An interaction between *time* and *treatment* (0 = FTY, 1 = DMF) |
| baseval_age | Age at baseline (in years) |
| baseval_sex | Gender at baseline (0 = female, 1 = male) |
| baseval_educ | Years of education at baseline |
| baseval_msdur | Duration of MS at baseline (in years) |
| baseval_relapses | Baseline number of relapses in the past 12 months |
| baseval_mstype | Baseline MS type (0 = Progressive Relapsing MS or Relapsing Remitting MS, 1 = Primary Progressive MS, 2 = Secondary Progressive MS) |
| baseval_prior_dmt_effic | Prior DMT efficacy at baseline (0 = No prior DMT, 1 = Low efficacy, 2 = Medium efficacy, 3 = High efficacy) |
| baseval_cardio | History of cardiovascular disease at baseline (0 = No, 1 = Yes) |
| baseval_diabetes | History of diabetes at baseline (0 = No, 1 = Yes) |
| penterid | An identifier of the MS centre |
| patid | An identifier of the patient |

## A4.3 |Balance diagnostics

Figure depicts covariate balance measured by standardized mean difference (SMD) in the cohort of 456 patients with complete baseline data before and after weighting. The inverse probability weights are based on the following baseline covariates: age, sex, MS type, years of education, disease duration, PDDS score, self-reported number of relapses in the past 12 months, prior DMT efficacy, history of cardiovascular disease, and history of diabetes.


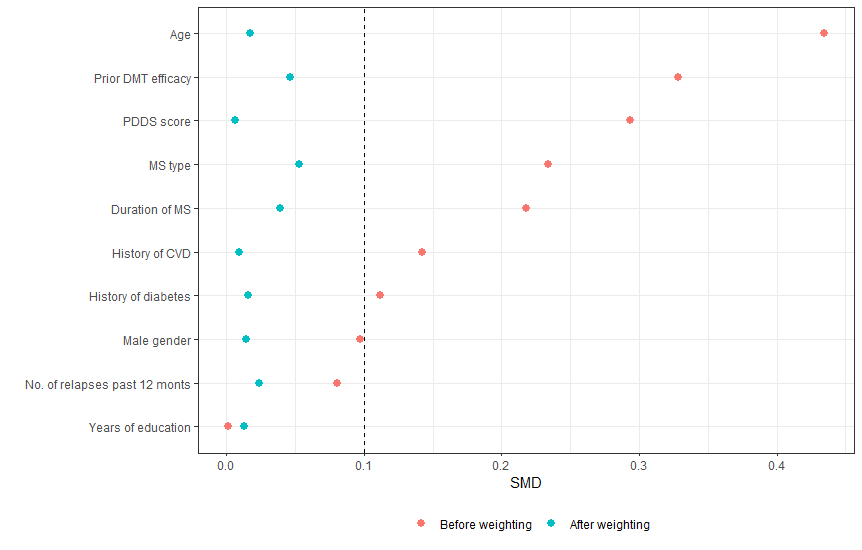


Figure 8 Covariate balance before and after weighting, as assessed with standardized mean differences (SMDs). An absolute SMD below 0.10 (dashed line) is considered satisfactory balance.

## A4.4 | Multilevel analysis in the complete cases

We fitted the model specified in section **A3.2** to the patients with complete baseline data (N = 456) to evaluate the treatment effect on a continuous scale and to quantify the presence of autocorrelation.

| **Parameter** | **Value** | **Standard error** | **P-value** |
| --- | --- | --- | --- |
| (Intercept) | 0.33 | 0.42 | 0.43 |
| tdays | $2.43\times{10}^{-4}$ | $0.95\times{10}^{-4}$ | 0.01 |
| time_x | $-0.64\times{10}^{-4}$ | $1.46\times{10}^{-4}$ | 0.66 |
| baseval_age | 0.03 | 0.01 | <0.01 |
| baseval_sex | 0.12 | 0.16 | 0.45 |
| baseval_educ | -0.09 | 0.02 | <0.01 |
| baseval_msdur | 0.02 | 0.01 | 0.02 |
| baseval_relapses | 0.39 | 0.07 | <0.01 |
| baseval_mstype | 0.76 | 0.09 | <0.01 |
| baseval_prior_dmt_effic | -0.03 | 0.08 | 0.71 |
| baseval_cardio | 0.30 | 0.14 | 0.03 |
| baseval_diabetes | 0.05 | 0.26 | 0.85 |

The treatment effect estimate was captured by the coefficient for *time_x* and indicated that DMF reduced PDDS progression by 0.02 point per year as compared to FTY (not statistically significant). The estimate for the range parameter was 14.515. Corresponding autocorrelation values are displayed in Figure **7** as a function of the time duration between successive visits**.**


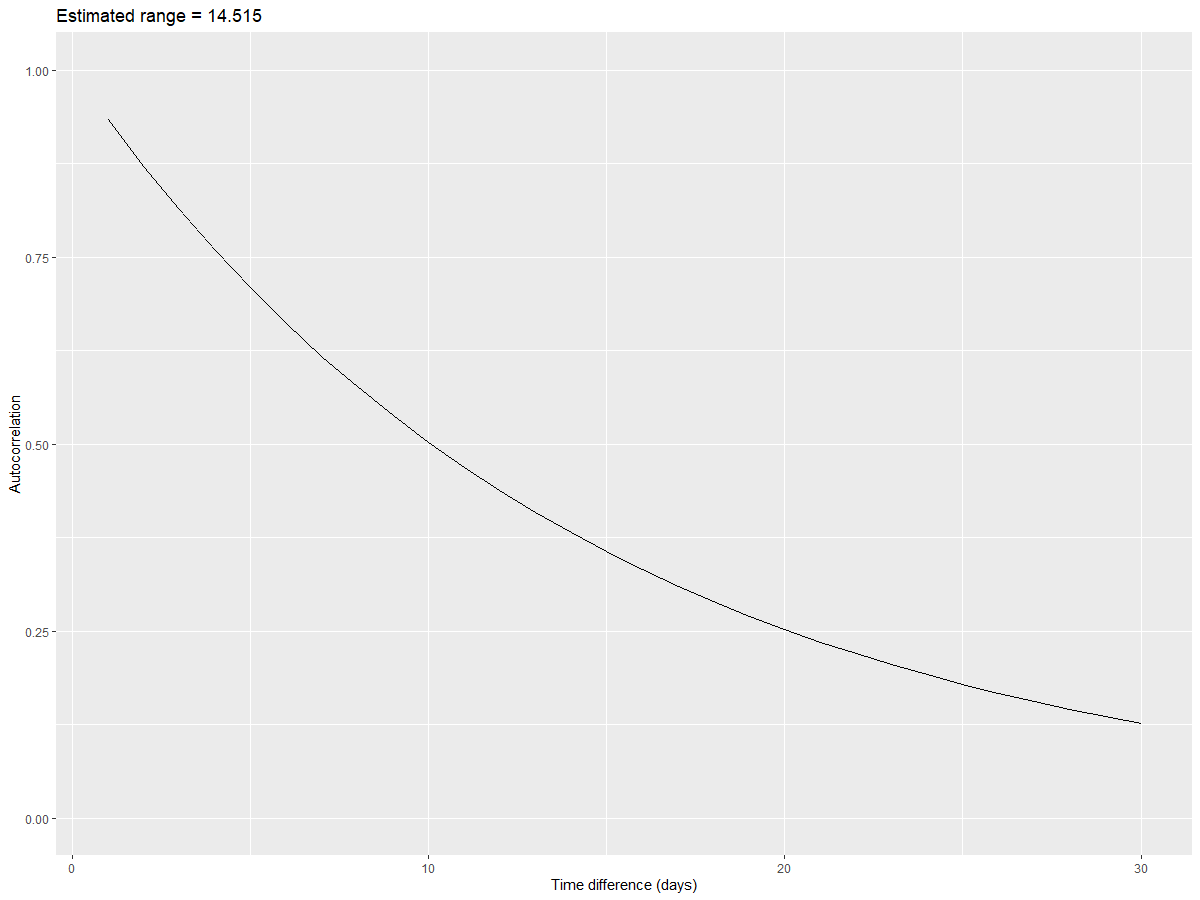


Figure 9 Autocorrelation values for the estimated range of 14.515.

## A4.5 | Recovered PDDS trajectories

Examples of observed and imputed PDDS trajectories with (a) LOCF, (b) rounding and (c) MLMI-PMM (for 4 of the 50 imputed trajectories).


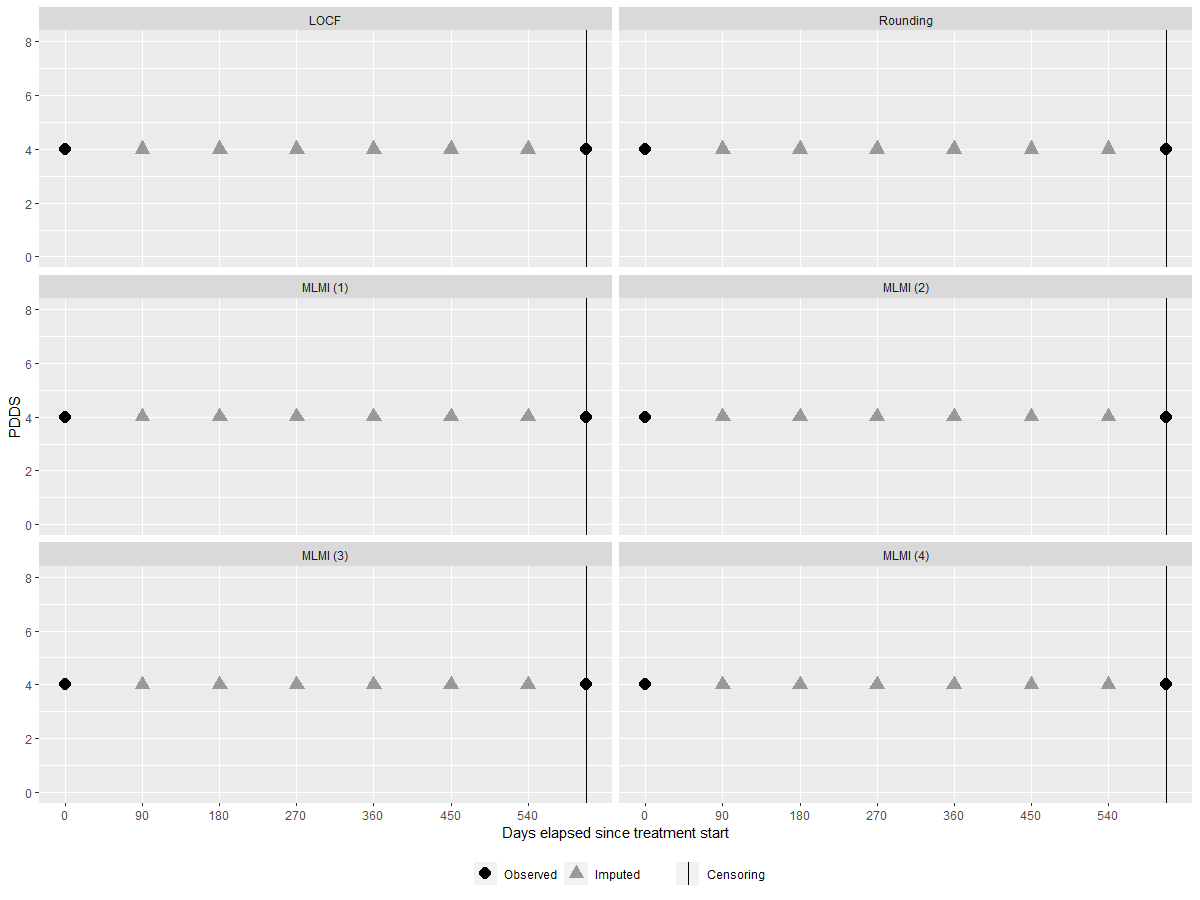


Figure 10 Recovered PDDS trajectory for a patient with 2 visits during a follow-up period of 608 days.


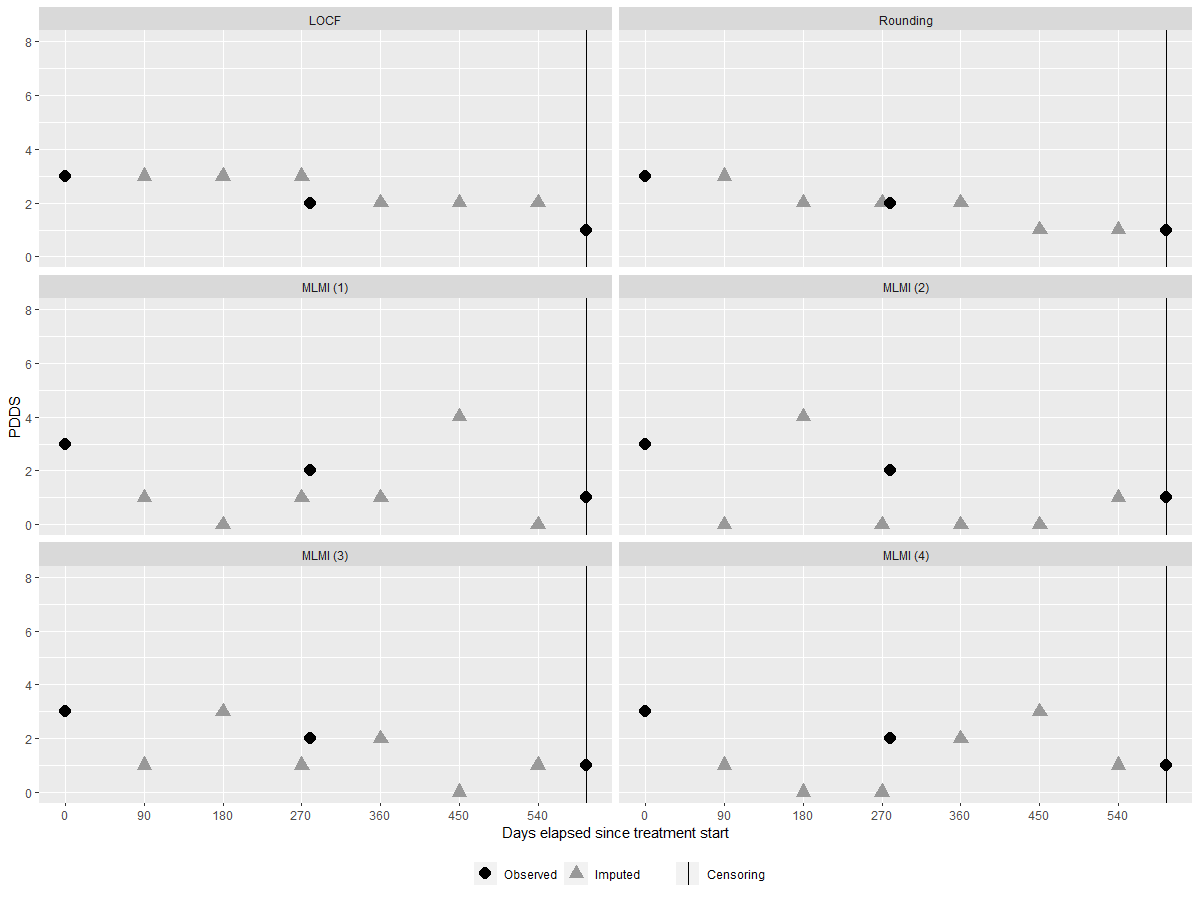


Figure 11 Recovered PDDS trajectory for a patient with 3 visits during a follow-up period of 595 days.


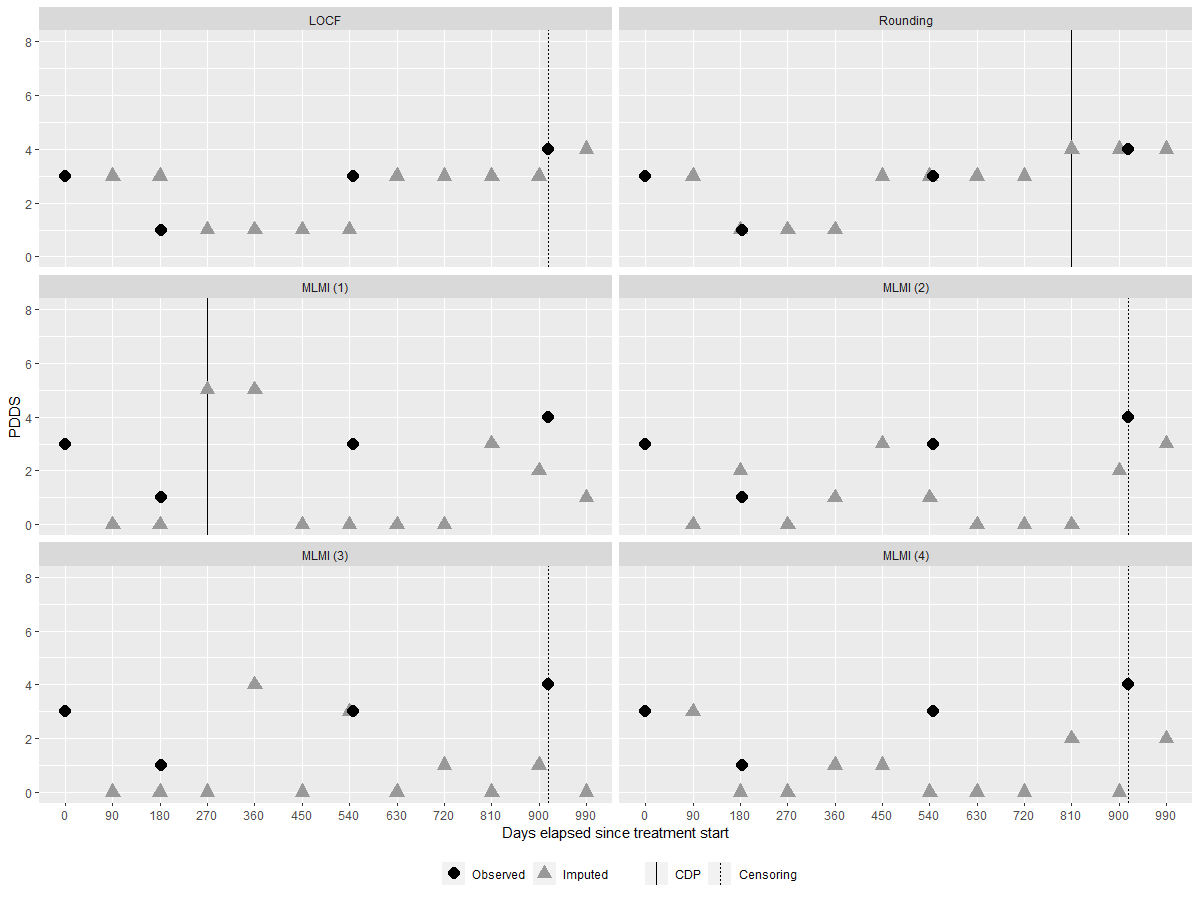


Figure 12 Recovered disease trajectory for a patient with 4 visits during a follow-up period of 917 days.


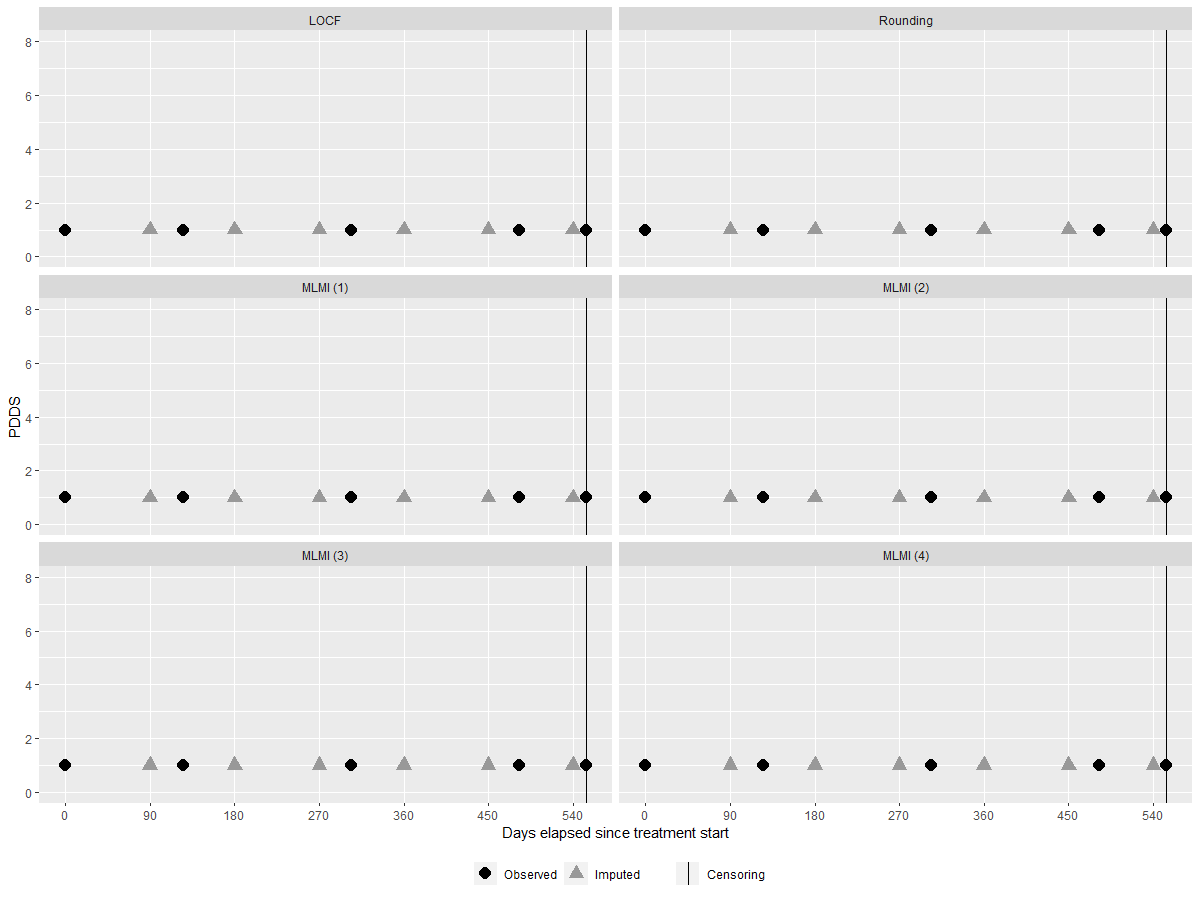


Figure 13 Recovered disease trajectory for a patient with 5 visits during a follow-up period of 554 days.

## A4.6 | Recovered survival data


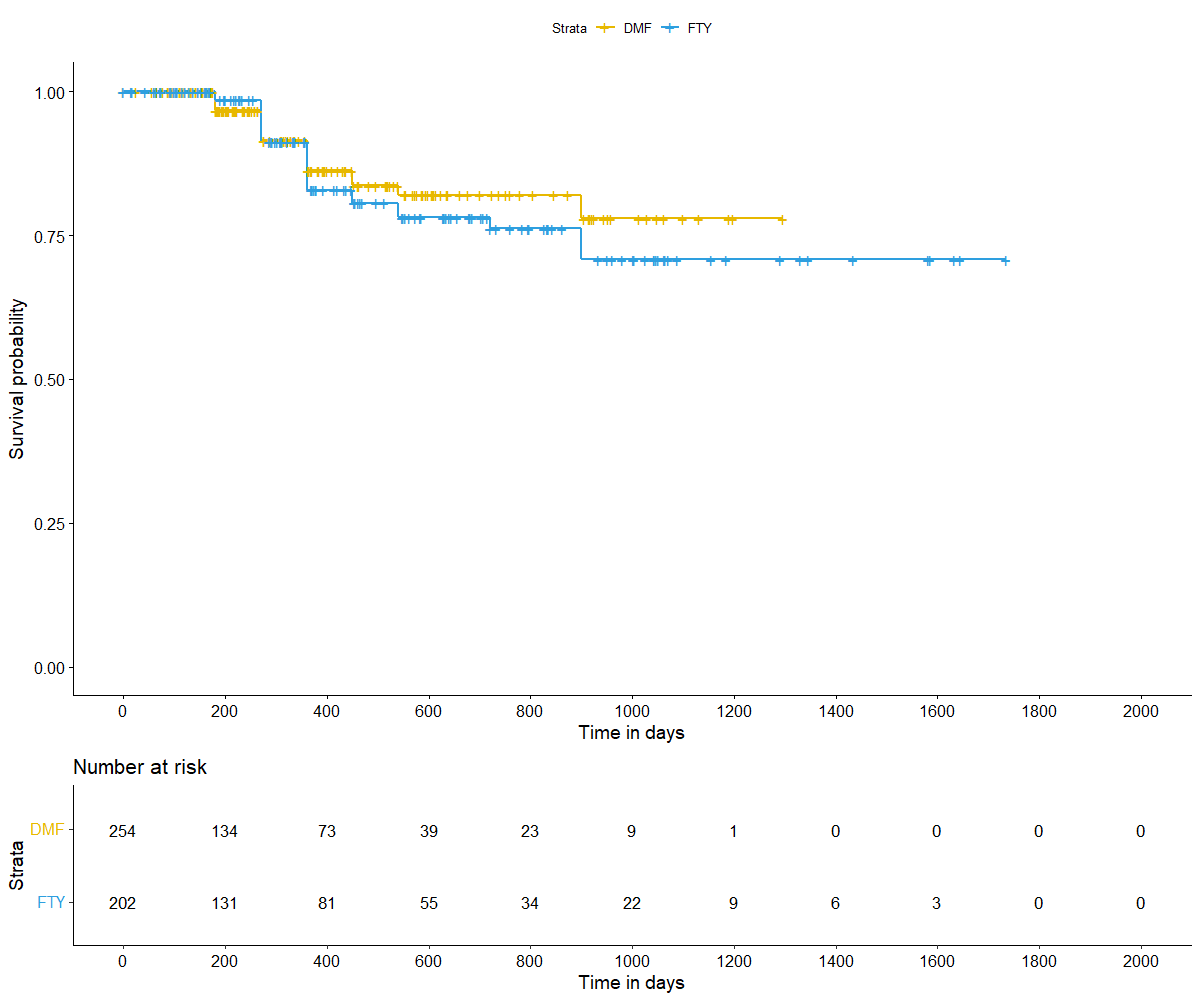


Figure 14 Estimated survival curve for the cohort of 456 patients with complete baseline data when adopting LOCF to recover the PDDS along a 3-month grid.


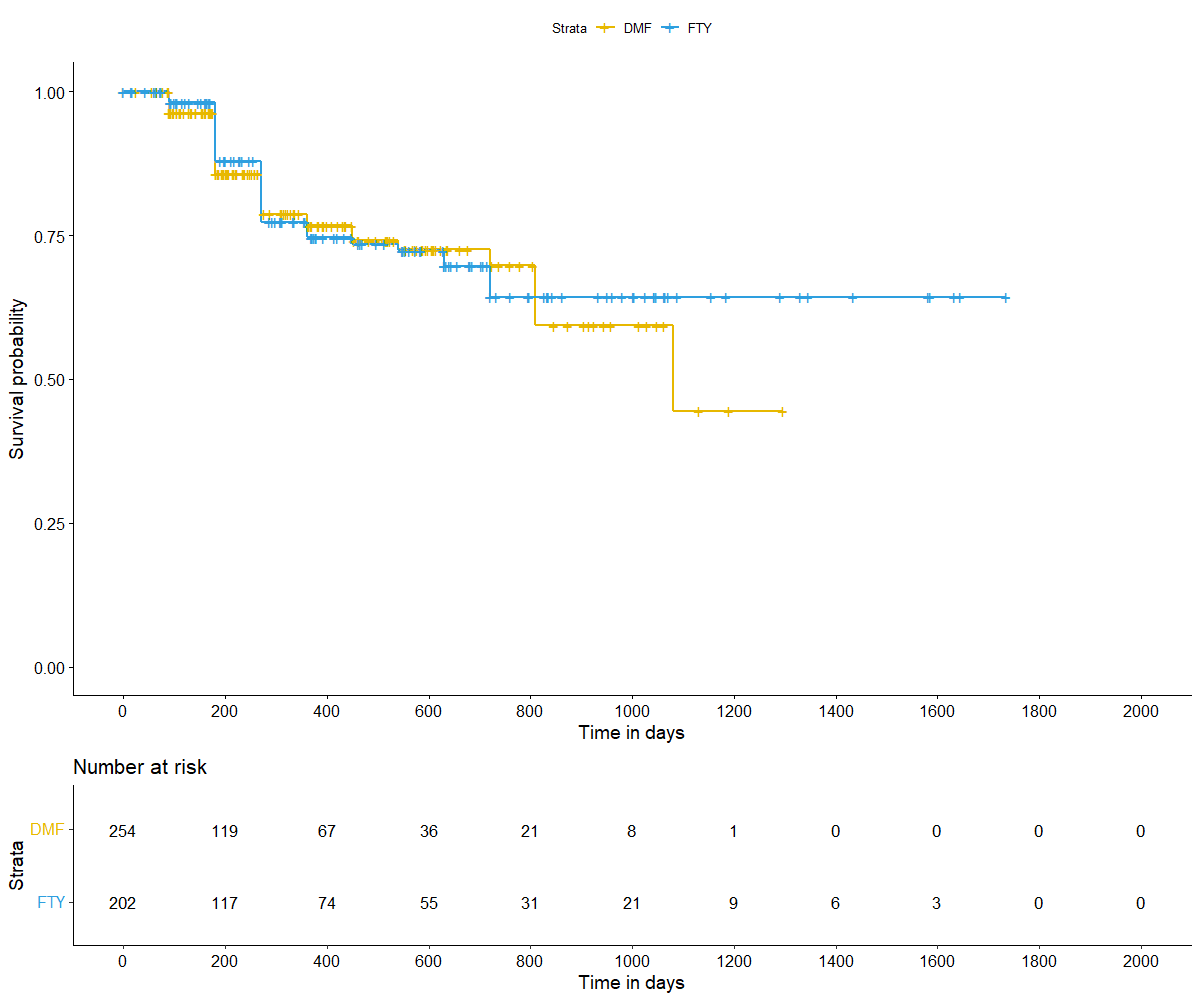


Figure 15 Estimated survival curve for the cohort of 456 patients with complete baseline data when adopting Rounding to recover the PDDS along a 3-month grid.


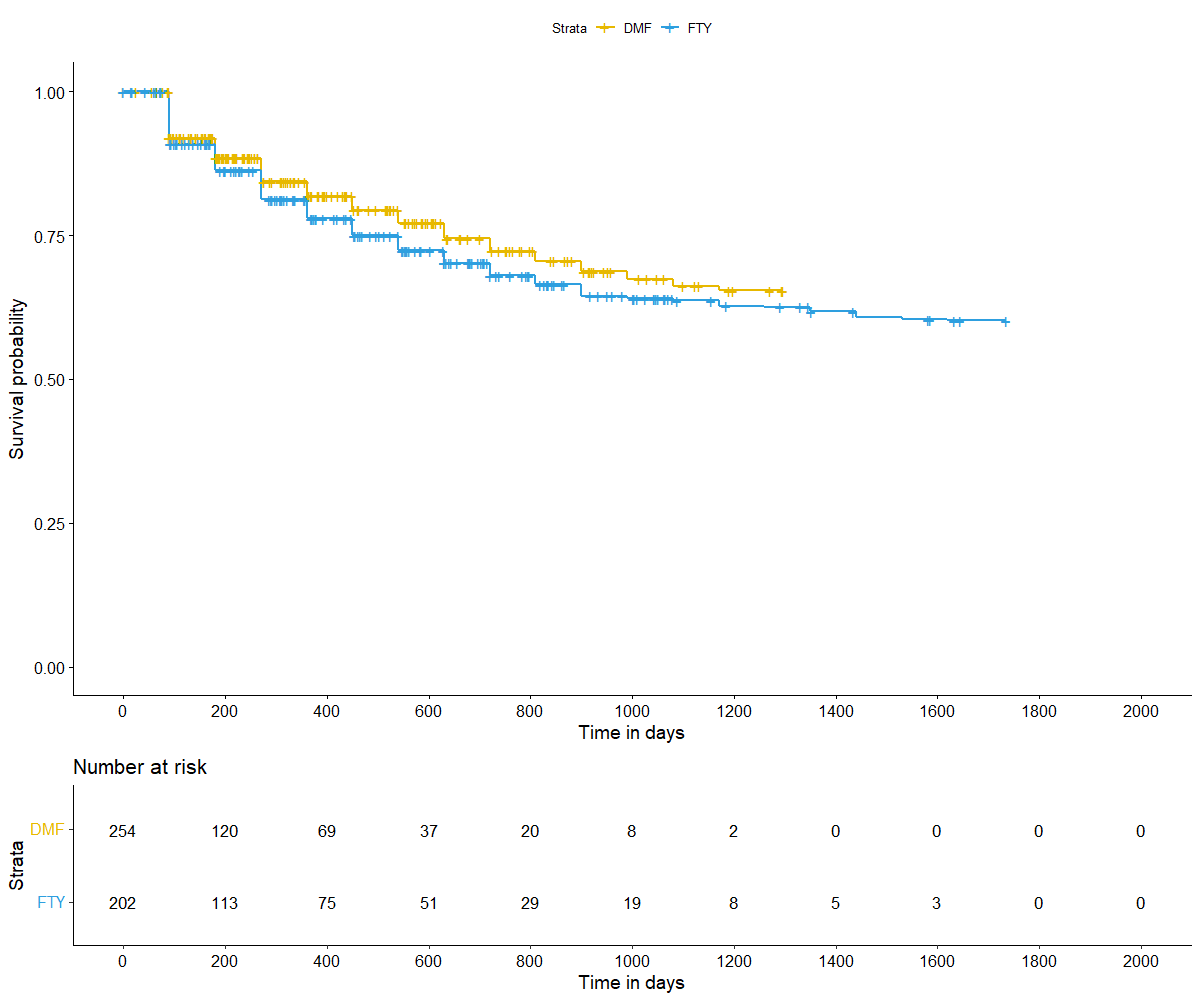


Figure 16 Estimated survival curve for the cohort of 456 patients with complete baseline data according to a 3-month imputation grid. Multilevel Multiple Imputation with rounded PDDS scores was used to generate 100 imputed datasets, which were analysed using a weighted Kaplan Meier analysis.


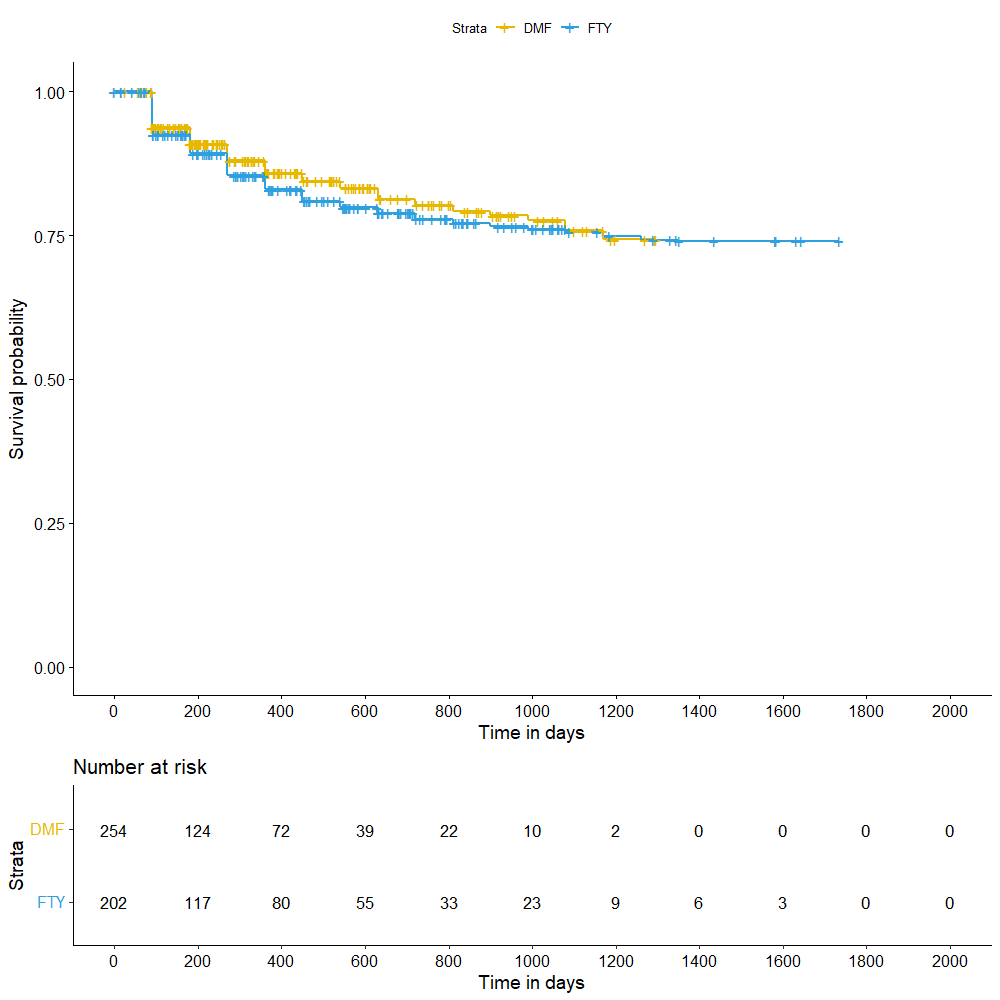


Figure 17 Estimated survival curve for the cohort of 456 patients with complete baseline data according to a 3-month imputation grid. Multilevel Multiple Imputation using Predictive Mean Matching was used to generate 100 imputed datasets, which were analysed using a weighted Kaplan Meier analysis.


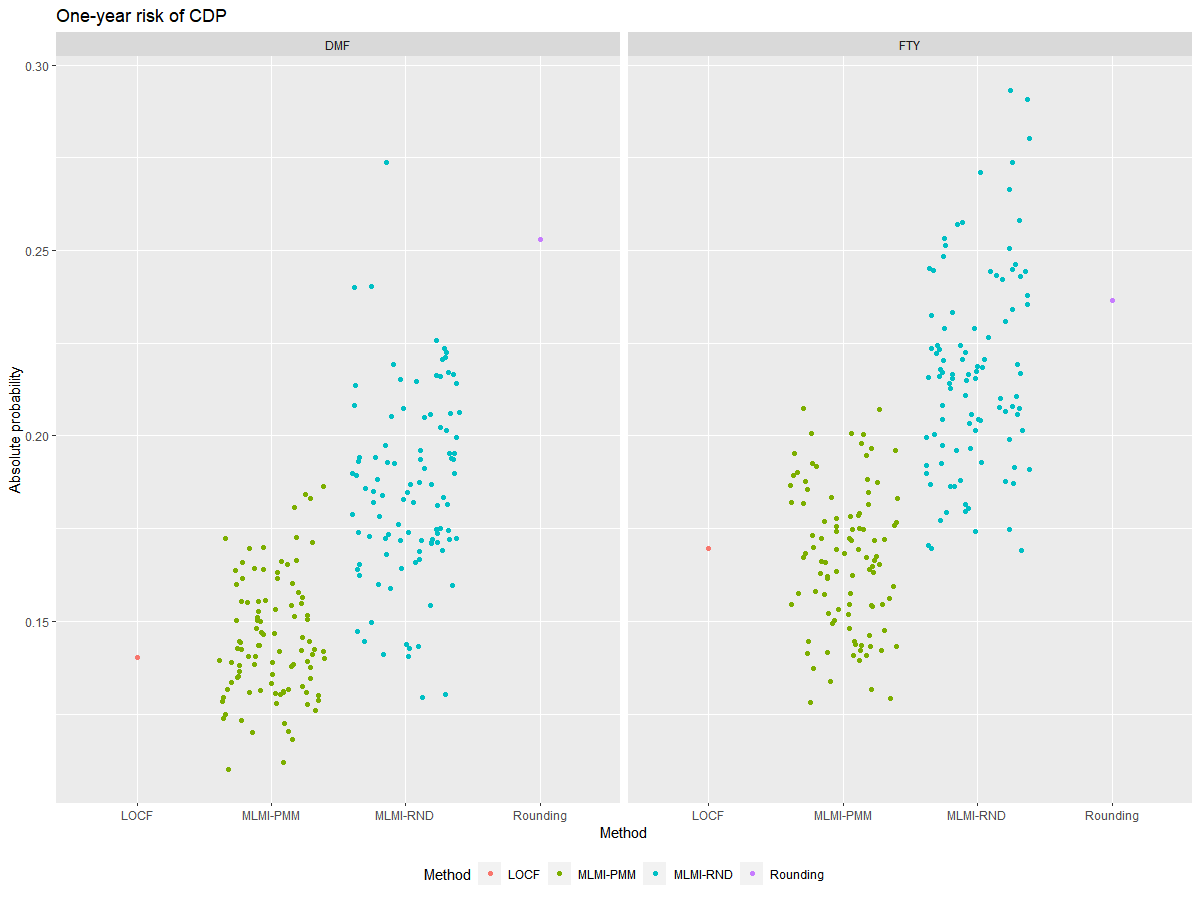


Figure 18 Cumulative probability of developing CDP within 365 days for an “average” individual. Survival data were derived from a 3-month imputation grid. Risk estimates are based on a Cox regression model that is adjusted for received treatment only. MLMI-RND = multilevel multiple imputation with rounded PDDS scores; MLMI-PMM = multilevel multiple imputation with predictive mean matching.

# References

1. Gold R, Kappos L, Arnold DL, Bar-Or A, Giovannoni G, Selmaj K, et al. Placebo-Controlled Phase 3 Study of Oral BG-12 for Relapsing Multiple Sclerosis. N Engl J Med. 2012 Sep 20;367(12):1098–107.
