# Supplementary material for: Methods for comparative effectiveness based on time to confirmed disability progression with irregular observations in multiple sclerosis
Source: Stat Methods Med Res. 2023 Jun 11;32(7):1284–99. doi: 10.1177/09622802231172032 (PMC10500950; doi:10.1177/09622802231172032)
Supplement: sj-zip-2-smm-10.1177_09622802231172032 - Supplemental material for Methods for comparative effectiveness based on time to confirmed disability progression with irregular observations in multiple sclerosis [file sj-zip-2-smm-10.1177_09622802231172032.zip › Simulation.pdf]

Explanation of the R code

```
source("R/dgm.r")
source("R/vis.r")
source("R/imp.r")
source("R/sim.r")
```

We first need to set up the data-generating mechanism to enable the generation of a non-randomized dataset with two treatments. By default, a total of 61 visits will be generated for each patient, with a frequency of one visit per month. The outcome at each visit (EDSS) is determined by the received treatment, the total treatment exposure time, and the age at baseline. In addition, random variation is added to the EDSS scores, with clustering by patient ID and by center ID.

```
simpars <- setup(delta_xt = -0.007)
```

The configuration above ensures that the active treatment will reduce deterioration in EDSS by 0.007 points each day (as compared to the control treatment). We can now simulate a dataset:

```
dat <- sim_data(simpars)
head(dat)
```

```
##   centerid patid trt      age time edss
## 1        1     1   0 42.75642    0  3.0
## 2        1     1   0 42.75642    1  3.5
## 3        1     1   0 42.75642    2  3.5
## 4        1     1   0 42.75642    3  3.5
## 5        1     1   0 42.75642    4  3.5
## 6        1     1   0 42.75642    5  4.0
```

So far, the simulated dataset does not contain any missing visits. We can introduce irregular visit patterns by informatively censoring certain vists from `dat`. Eight different informative censoring functions are available from `R/dgm.r`. We will consider `censor_visits_7()`, which introduces the following visit pattern:

```
plot_md_pattern(simpars, censor_visits_7)
```

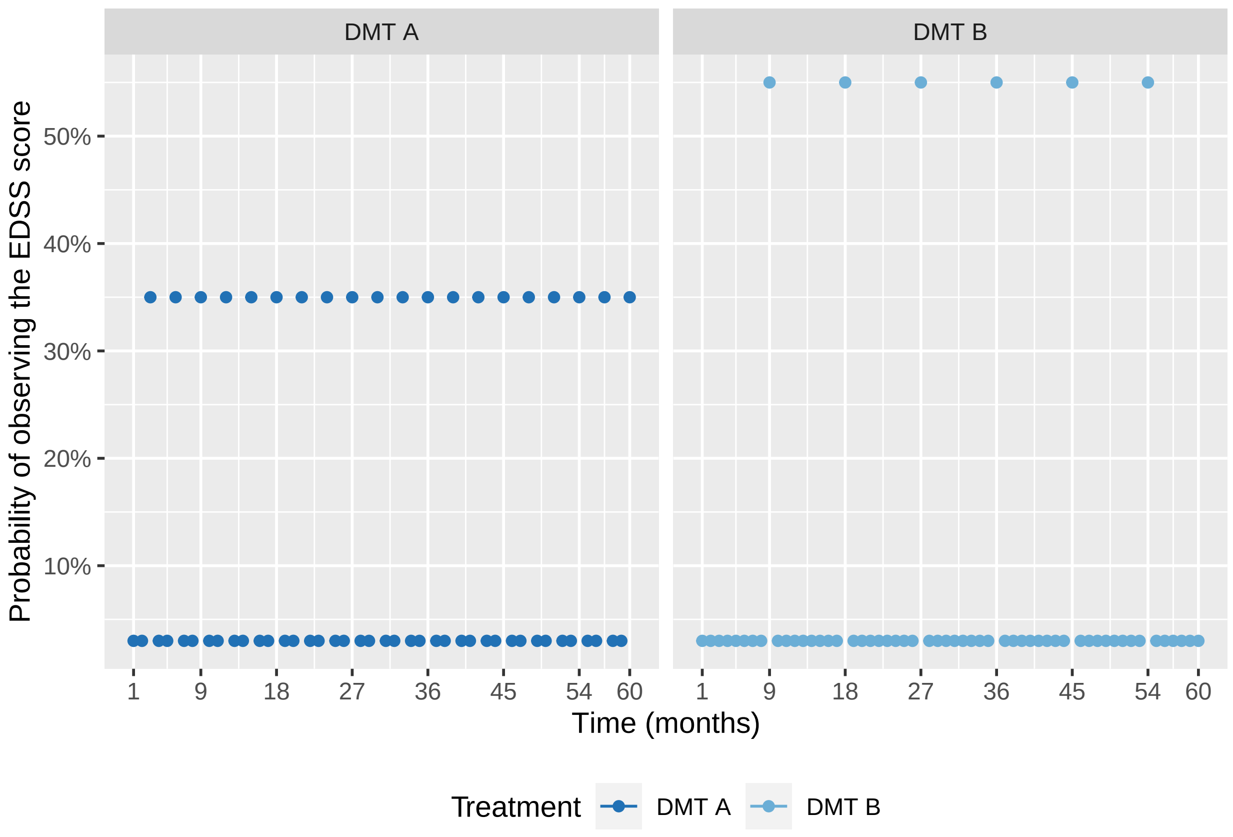

We can apply the informative visit process as follows:

```
misdat <- censor_visits_7(dat)
subset(misdat, patid == 1)
```

```
##    centerid patid trt      age time edss
## 1         1     1   0 42.75642    0  3.0
## 10        1     1   0 42.75642    9  3.5
## 17        1     1   0 42.75642   16  3.5
## 30        1     1   0 42.75642   29  4.0
## 34        1     1   0 42.75642   33  4.0
## 37        1     1   0 42.75642   36  4.0
## 46        1     1   0 42.75642   45  3.5
## 52        1     1   0 42.75642   51  3.5
## 55        1     1   0 42.75642   54  3.0
```

The observed EDSS trajectory of patient with `patid=1` is then as follows:

```
plot_example_trajectory(misdat, sel_patid = 1)
```

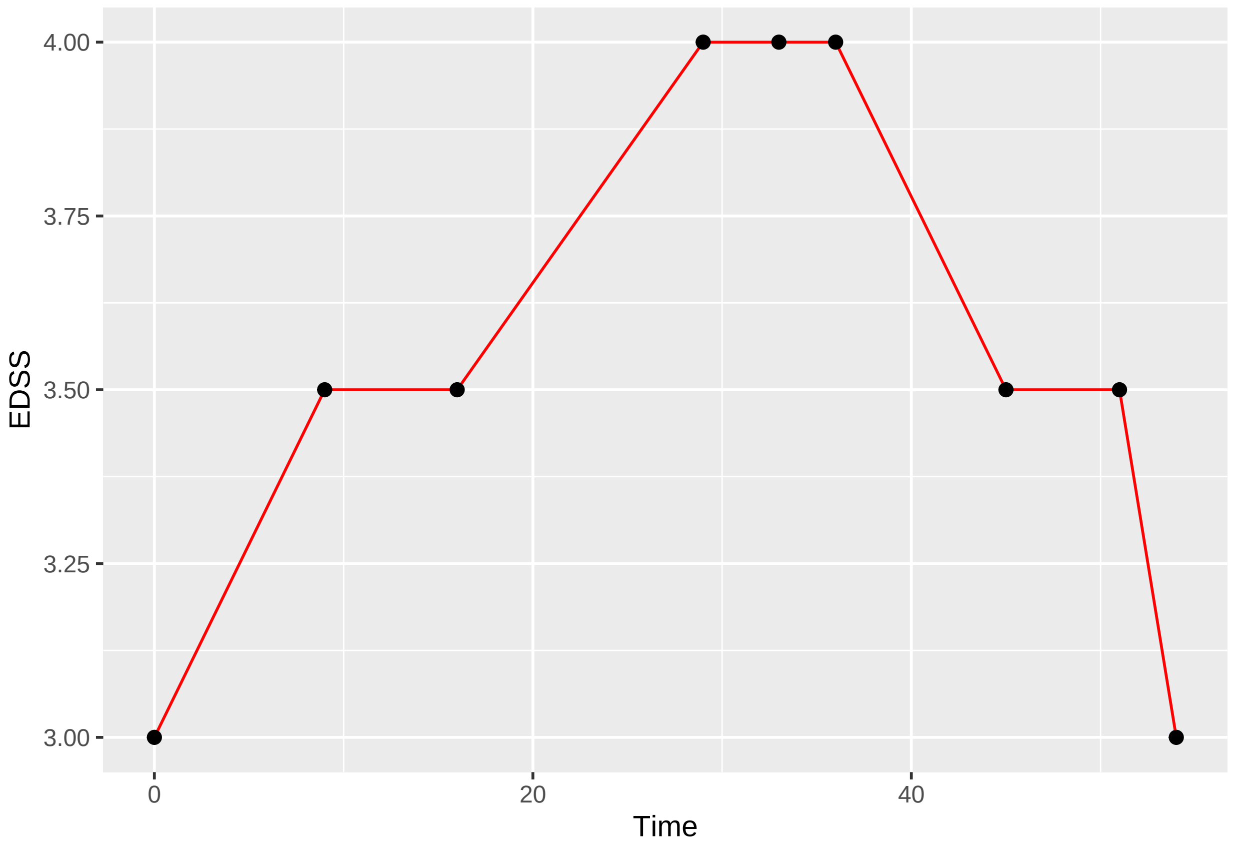

We can also visualize the age distribution across the treatment groups:

```
plot_dens_x(misdat, x_var = "age", x_label = "Age")
```

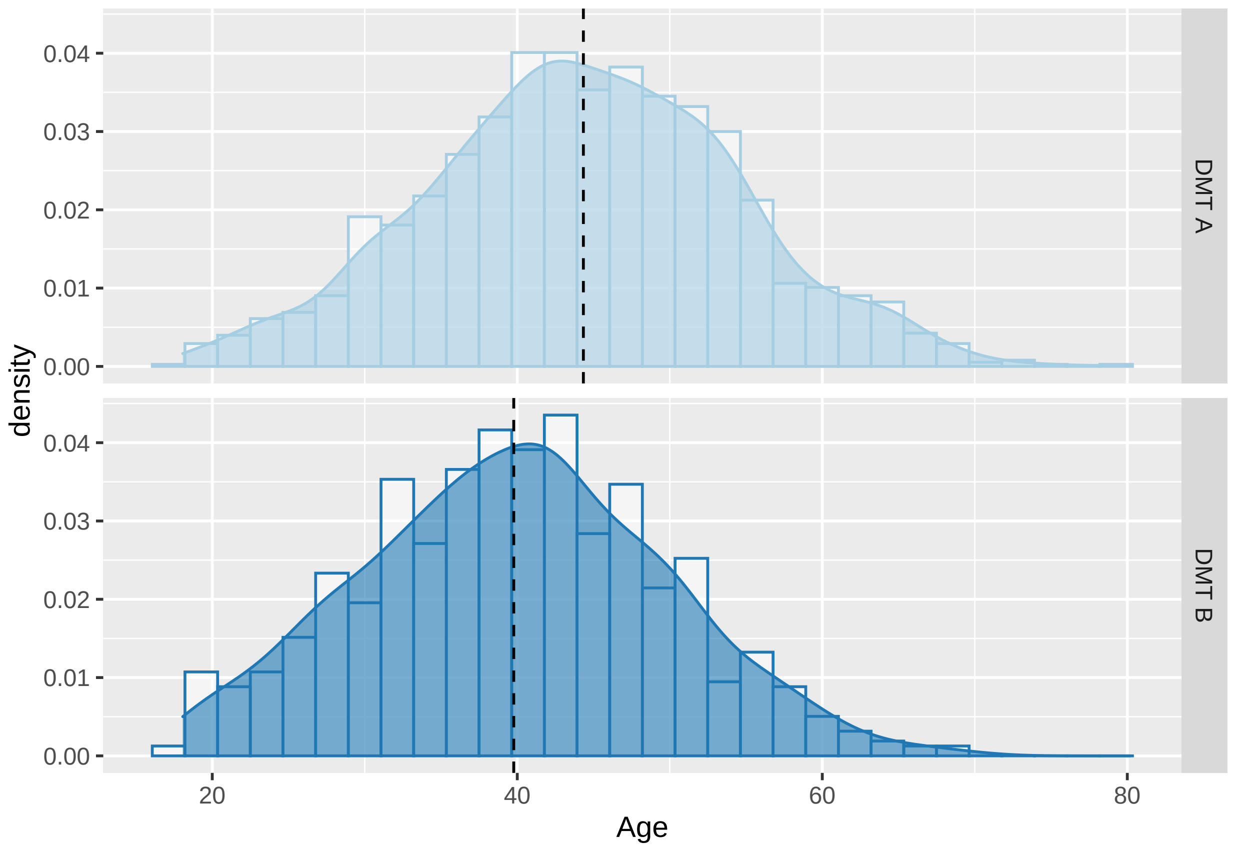

We can visualize the total follow-up for each center:

```
plot_max_fup(misdat)
```

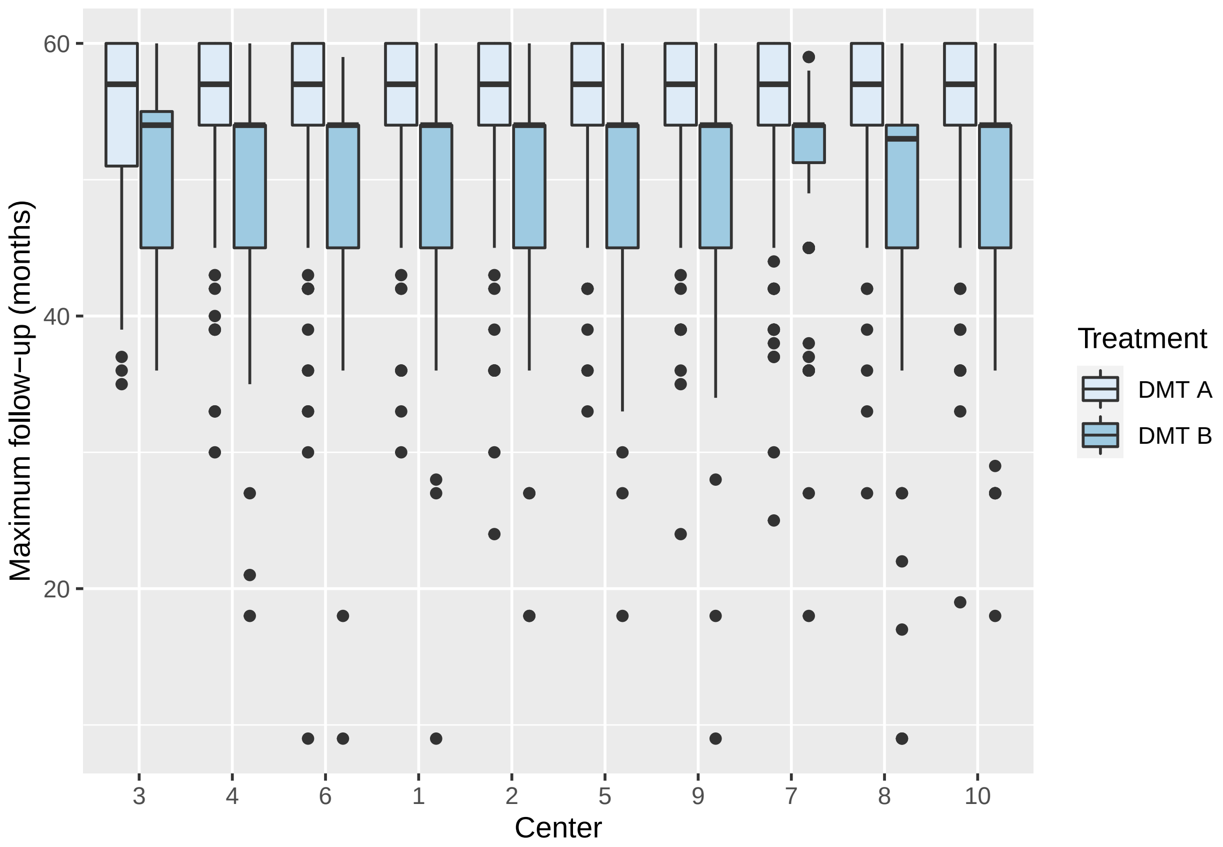

We can now generate an imputed dataset using the `mice` software package. We will only generate imputed values on a 3-month grid.

```
imptimes <- seq(0, 60, by = 3)
imp <- impute(misdat, times = imptimes, maxit = 1)

# Visualize the imputed trajectory of one patient
plot_imputed_trajectories(imp, sel_patid = 1)
```

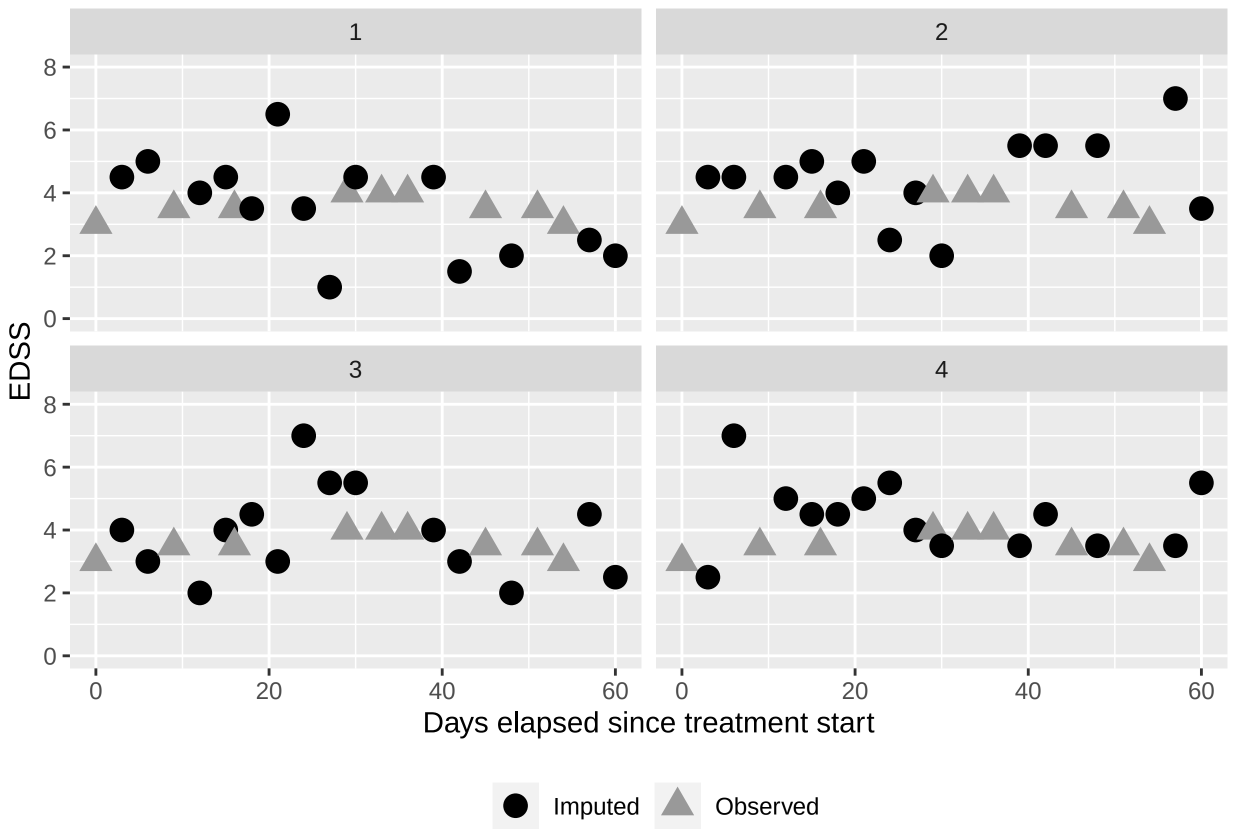

Finally, we can run a simulation study to assess the ability of the imputation model to recover the missing EDSS scores and to estimate the treatment effect. Since no Gibbs sampler is needed in this case, we implemented a faster version that bypassess the need of MICE and generates 10 imputed datasets simultaneously.

```
sim <- run_sim(simpars, censor_visits_7, seed = 101)
```

```
##   |                                                                              |
```

```
sim
```

```
##   delta_xt sim_id system_name system_machine                    R_version
## 1   -0.007    101     Windows         x86-64 R version 4.0.2 (2020-06-22)
## 2   -0.007    101     Windows         x86-64 R version 4.0.2 (2020-06-22)
## 3   -0.007    101     Windows         x86-64 R version 4.0.2 (2020-06-22)
## 4   -0.007    101     Windows         x86-64 R version 4.0.2 (2020-06-22)
## 5   -0.007    101     Windows         x86-64 R version 4.0.2 (2020-06-22)
##                      method  est_logHR    est_HR est_HR_CIl est_HR_CIu window
## 1                 Reference -0.3707462 0.6902191  0.5994803  0.7946924      3
## 2                      LOCF -0.3376605 0.7134375  0.6132510  0.8299913      3
## 3                  Rounding -0.3172183 0.7281717  0.6215991  0.8530162      3
## 4              LME-CE (PMM) -0.3307769 0.7185456  0.6256175  0.8257436      3
## 5 LME-CE (EDSS conversion) -0.2477846 0.7809786  0.6853083  0.8901705      3
##   confirmation nobs_imputed      rmse
## 1            1            0 0.0000000
```

```
## 2            1        29516 0.6190101
## 3            1        29516 0.5496179
## 4            1        29516 0.4930344
## 5            1        29516 0.5129522
```
